# Tackling toxicity in Arabic social media through advanced detection techniques

**DOI:** 10.1038/s41598-025-25879-4

**Published:** 2025-11-21

**Authors:** Loay Hatem, Ahmed Omar, Abdelmgeid A. Ali, Heba Mamdouh Farghaly

**Affiliations:** 1 Computer Science Department, Faculty of Science, Minia University, Minya, Egypt; 2 Artificial Intelligence Program, Faculty of Computers and Artificial Intelligence, Minia National University, Minya, Egypt

**Keywords:** Arabic NLP, Arabic toxicity classification, Online social network (Twitter), Machine learning (ML), Transfer learning (TL), Transformers (Arabic BERT models), Engineering, Mathematics and computing

## Abstract

Online social networks are currently the most widely utilized interactive media for interpersonal communication, emotional expression, and information sharing. Despite the helpful and fascinating content, unfortunately, inappropriate or abusive content, such as toxicity, hate speech, and insults, can occasionally be shared on social networks. Any kind of online abuse, including but not limited to cyberbullying, discrimination, abusive language, profanity, flames, hate speech, and harassment, is considered toxic content. While there has been little effort in the Arabic language, the majority of toxicity detection attempts have focused on English text. In this work, we constructed a standard Arabic dataset that can be used for toxicity and abuse detection on OSNs. The proposed dataset has been annotated by the experts of five native and fluent Arabic speakers and linguists. To evaluate the performance of our dataset, we conducted a series of experiments by using sixteen machine learning algorithms, the FastText model, and seven transfer learning architectures to compare the performance. Furthermore, we used four word embedding techniques (bag of words (BOW), term frequency–inverse document frequency (TF-IDF), FASTTEXT, and bidirectional encoder representations from transformers (BERT)). Our experimental results demonstrated that the fine-tuned MARBERTv2 model with BERT embedding outperforms the other models, achieving an F1-score of 92.43% and an accuracy of 92.21%. Notably, this study highlights the importance of addressing toxicity on social media platforms, considering diverse languages and cultures. This signifies a significant breakthrough in the classification of toxic tweets in Arabic.

## Introduction

Warning: This paper addresses the problem of offensive or upsetting Arabic language. Therefore, it may contain some examples that include offensive or vulgar words. These examples do not reflect the authors’ perspective in any way.

The emergence of social media has expanded the reach of the internet. Online social networks (OSNs) have emerged as the most widely used form of interactive communication and sharing platform. A variety of data formats, including text, audio, pictures, and video, are included in the phrase “communication”.

Textual data is the most widely shared content on social media, appearing in tweets, comments, and responses^1,2^. Owing to the vast volume of text accessible on OSNs, text mining has emerged as a fascinating area of study. On the Internet, there are over 900 social networking platforms available^1^. Twitter, one of the most widely used social networking platforms, ranks twelfth globally with over 666 million active users^3^. Users spend a substantial amount of time sharing content through tweets, retweets, and replies. Every minute, an estimated 456,000 tweets are posted^4^, and monthly, users generate enormous volumes of multimedia and text-based content, including photos, videos, and links.

Social media allows people from various backgrounds to connect with like-minded individuals, fostering mutually beneficial networks. These users have diverse religious, ethnic, political, and ethical backgrounds^5^. People may communicate with each other and express themselves, their cultures, and their opinions via social media. It makes sense that differences in opinion might spark conversations. These debates often grow ugly, and some end up as online fights where one side may use toxic language or offensive and abusive comments and remarks^6^.

This type of behavior damages users’ social and psychological well-being and decreases their propensity to trust online social media. Hence, a remedy needs to be discovered before this expanding hate takes hold and ruins the lives of individuals, families, and communities^7^. The research community has also attempted to identify online toxicity and abusive behavior^8^.

Toxic remarks are words, phrases, or declarations that convey or suggest disdain for other people^9^. These remarks make one feel dehumanized or insulted. Toxic material is becoming more prevalent in communities, regardless of whether the people involved are acquainted. Social media platforms have tried to block harmful information, but their efforts have largely failed and have had unanticipated results. The effectiveness of these platforms’ moderation may be skewed by their economic objectives or by political and regulatory considerations^5^.

Most detrimental classification attempts have concentrated on the English language. There have not been many attempts to achieve the same results for the Arabic language, which has several difficulties. Arabic is now the fourth most commonly used language online, and its content has experienced significant growth in recent years. Arabic content currently constitutes more than 3% of all internet content, securing its seventh overall rank. The Arabic language is rich in content, with approximately 226 million Arabic internet users worldwide^10^. The Arabic language contains many dialects, such as Modern Standard Arabic (MSA), Egyptian, Gulf, Hassaniya, Levantine, Maghrebi, Mesopotamian, Sudanese, Yemeni, Hijazi Arabian, and Maltese, but MSA is the most widely used dialect of Arabic and has been specifically modified to standardize speech and writing^11^.

Social network users generate one-third of the poor-quality Arabic content that is available online^10^. Arabic datasets are rarely supported and have seen limited utilization in research. The analysis and classification of Arabic text are therefore gaining increasing importance. As Arabic becomes more prevalent on the internet, the challenges associated with its analysis, coupled with the scarcity of research on Arabic toxicity detection, underscore the need for further investigations in this area.

The research community in Arabic text classification faces a variety of challenges, including diacritical marks (i.e., a small sign that precedes a letter to indicate a change in the corresponding sound or to distinguish between homonyms). Diacritical marks, also known as diacritics or accents, are symbols added to letters to indicate specific pronunciations or meanings. For example, consider the words “درس” and “drs”: when the diacritical fatha mark is applied to the first letter and the diacritical sukun mark is applied to the second letter (“دَرْس”), the word means “lesson”. However, if the diacritical mark fatha is also applied to the first and second letters (“دَرَس”), the word means “studied”. Furthermore, when the diacritical damma mark appears over the first letter and the kasra mark appears under the second letter (“دُرِس”), the word “has been studied”^12^.

The proposed method involves building a new Arabic dataset and finding the best technique among the machine and transfer learning algorithms for Arabic text classification in terms of improving social media network surfing life.

The main contributions of this paper are as follows:

- We constructed a standard Arabic dataset for toxicity assessment via manual annotation techniques.
- The corpus contains a variety of Arabic dialects, with more than 15 Arabic dialects.
- The size of the dataset is almost 50,000 Arabic-balanced tweets, which is large enough compared with previous studies.
- We trained sixteen machine learning algorithms, the FastText model (Default Form), and seven transfer learning algorithms for Arabic toxicity detection in OSNs (especially Twitter).

The remaining sections of this paper are organized as follows: Sect. 2 presents the most recent related work on toxicity and abusive detection. Section 3 describes the models used in the classification process and their backgrounds. Section 4 reviews the methodology, including dataset construction, preprocessing stages, annotation, feature representation, and the classification model. Section 5 presents, discusses, examines, and compares the experimental results and discussions. Section 6 represents the limitations of this study. Finally, Sect. 7 concludes the conclusion of our study and determines our future work.

## Related work

Abuse detection has long been an area of growing research interest in the past few years. While there have been some attempts to address this problem in online social networks, there is a surprising lack of attention devoted to the Arabic language. Cyberbullying, discrimination, abusive language, vulgarity, flame, hate speech, and harassment are only a few examples of online abuse themes that have been considered toxic in literature.

The following paragraphs present some of these efforts and are summarized in Table 1.

Harbaoui, A. et al.^13^ utilized three datasets: an Arabic dataset of 32,000 comments in MSA and various dialects collected from the Aljazeera News Channel website, and two English datasets with 12,896 samples from the Formspring.me forum and 6,580 samples from the Impermium dataset^14–16^. All datasets employed Sentence Embeddings Generator (SEG) and Pre-trained Language Models (PLMs) for feature extraction, with labels categorized as Bully or Non-Bully. Classifiers included Convolutional Neural Networks (CNN)-Attention, Universal Sentence Encoder (USE) combined with Logistic Regression (USE + LR), USE combined with XGBoost (USE + XGB), and fine-tuned RoBERTa (FT). The best-performing models were USE + LR for the Arabic dataset (Precision 34%, Recall 81%, F1-score 48%), USE + XGB for Formspring.me (Precision 36%, Recall 75%, F1-score 49%), and RoBERTa (FT) for Impermium (Precision 72%, Recall 86%, F1-score 78%).

Lee, N. et al.^17^ employed three datasets: 12,698 Arabic tweets in MSA and various dialects from Twitter, 40,429 Korean comments from NAVER News and YouTube, and 150,000 English tweets and posts from Twitter, Reddit, and various hate-oriented websites (HateSites)^18–20^. All datasets used sentence-transformer embeddings such as Sentence BERT (SBERT) and were labeled as Hate or Non-Hate. Classifiers included AraBERTv0.2-Twitter_base_, KcELECTRA_base_-v2022, and BERTweet_base_. The best-performing models were AraBERTv0.2-Twitter_base_ for the Arabic dataset (Precision 84%, Recall 80%, F1-score 82%), KcELECTRA_base_-v2022 for the Korean dataset (Precision 83%, Recall 80%, F1-score 81%), and BERTweet_base_ for the English dataset, yielding Precision, Recall, and F1-scores of 86% each.

Mazari, A. C. and Kheddar, H^21^. developed an Arabic dataset in the Algerian dialect comprising 14,150 comments labeled as hate speech, offensive language, and cyberbullying, collected from Facebook, YouTube, and Twitter. They applied various feature representations and classifiers, including TF-IDF with Multinomial Naïve Bayes (MNB, F1-score 73.6%, Accuracy 66.3%) and Stochastic Gradient Descent (SGD, F1-score 72.3%, Accuracy 71.6%), Word2Vec embeddings (skip-gram) with MNB (F1-score 68.2%, Accuracy 53.4%) and Support Vector Classifier (SVC, F1-score 63.4%, Accuracy 55.7%), Word2Vec (CBOW) with SGD (F1-score 72.4%, Accuracy 56.9%), and FastText embeddings with Bidirectional Gated Recurrent Units (Bi-GRU), which achieved the best performance (F1-score 75.8%, Accuracy 73.6%).

Harrag, F. et al.^22^ utilized an Arabic dataset in MSA and various dialects, originally proposed by^23^, consisting of 3,353 tweets collected from Twitter. The dataset was annotated with hate categories such as race, gender, religion, ethnicity, and other attributes (targets). Three experiments were conducted using classifiers including AraBERT and AraGPT2. The best results were achieved in the second experiment using AraBERT with a maximum sequence length of 512, batch size of 6, and 4 training epochs, yielding Precision 78%, Recall 81%, F1-score 78%, and Accuracy 81%.

Alrashidi, B. et al.^23^ utilized a dataset in MSA and Arabic dialects, originally proposed by^24^, consisting of 3,353 tweets collected from Twitter. The dataset was annotated with five attribute categories: Directness, Hostility, Target, Group, and Annotator Reaction, each with multiple labels. For machine learning, TF-IDF was used with Support Vector Machines (SVM) and Naïve Bayes (NB). Deep learning approaches employed AraVec embeddings with CNN and LSTM classifiers, while several PLMs including MARBERT, ArabicBERT, CAMeLBERT, QARiB, and AraBERTv0.2 were incorporated using the NLPaug library^25^. Multi-task learning (MTL) architectures combined MARBERT with Long Short-Term Memory (LSTM), CNN, Bidirectional Long Short-Term Memory (BiLSTM), or their combinations. The best performance per attribute was as follows: Directness – MARBERT (Precision 68%, Recall 65%, F1-score 65%, Accuracy 67%); Hostility – MARBERT (Precision 45%, Recall 46%, F1-score 44%, Accuracy 46%); Target – CAMeLBERT (Precision 81%, Recall 82%, F1-score 82%, Accuracy 81%); Group – MTL with MARBERT + BiLSTM + CNN (F1-score 91%, Accuracy 89%); Annotator Reaction – CAMeLBERT (Precision 56%, Recall 57%, F1-score 56%, Accuracy 57%).

Al-Hassan, A. and Al-Dossari, H^26^. constructed an Arabic dataset in dialectal Arabic comprising 11,000 tweets collected from Twitter, annotated into five categories: religion, racism, sexism, general hate speech, and non-hate. For feature representation, TF-IDF and Keras Embedding were applied. Classification was performed using machine learning (SVM) and deep learning models (LSTM, CNN, Gated Recurrent Unit (GRU), and various ensemble models). The best performance was achieved by the ensemble model combining CNN and LSTM, with Precision 72%, Recall 75%, and F1-score 73%.

Badri, N. et al.^27^ utilized four Arabic datasets: the first three were originally proposed by^14,24,28,29^. The first dataset, in the Egyptian dialect, consisted of 1,101 comments collected from Twitter. The second dataset, in the Tunisian dialect, comprised 6,477 comments, with 6,040 sourced from^28^ (Facebook and YouTube) and 437 collected from Facebook by the authors. The third dataset, in the Lebanese dialect, included 9,200 comments collected from Twitter. Additionally, a fourth dataset was created by merging the previous three and adding new comments, resulting in 23,033 comments in Egyptian, Tunisian, and Lebanese dialects from Facebook, YouTube, and Twitter. All datasets were labeled as normal, hate, or abusive. Feature representations included AraVec, FastText, and TF-IDF. Deep learning models were CNN and BiGRU combined with AraVec and FastText embeddings, while machine learning approaches included LR and Random Forest (RF) with TF-IDF features. The best-performing models were: Egyptian dataset: CNN-BiGRU + FastText and CNN-BiGRU + FastText + AraVec (Precision 64%, Recall 63%, F1-score 63%); Tunisian dataset: RF (81% for Precision, Recall, and F1-score); Lebanese dataset: CNN + FastText + AraVec (Precision 79%, Recall 75%, F1-score 77%); merged dataset: CNN-BiGRU + FastText + AraVec (Precision 82%, Recall 81%, F1-score 82%).

Husain, F. and Uzuner, O^30^. employed four Arabic datasets originally proposed by^14,28,29,31^, each covering a different regional dialect. The Egyptian dataset comprised 1,100 tweets; the Tunisian dataset included 6,024 tweets; the Levantine dataset contained 5,846 tweets; and a multidialectal dataset consisted of 10,000 tweets. All datasets were collected from Twitter and annotated with binary labels: offensive or not offensive. Pre-trained models were evaluated across dialects, with AraBERT consistently achieving the best performance. For the Egyptian dataset, AraBERT achieved 78% for Precision, Recall, F1-score, and Accuracy. For the Tunisian dataset, Precision 79%, Recall 80%, F1-score 80%, and Accuracy 81% were obtained. On the Levantine dataset, AraBERT achieved Precision 86%, Recall 87%, F1-score 87%, and Accuracy 87%, and the same performance was achieved on the multidialectal dataset (Precision 86%, Recall 87%, F1-score 87%, Accuracy 87%).

Masadeh, M. et al.^32^ utilized an Arabic dialect dataset originally proposed by^33^, consisting of 6,164 tweets collected from Twitter, annotated with binary labels: Hate or Not Hate. Feature representations included BOW, TF-IDF, Word2Vec, and BERT-based embeddings. A range of machine learning and deep learning classifiers were evaluated, including Gradient Boosting, K-Nearest Neighbors (KNN), LR, NB, Passive Aggressive Classifier (PAC), SVM, and PLMs (AraBERT, AJGT-BERT) with different train-test splits. The best performance was achieved using AraBERT with an 80/20 train-test split, yielding Precision, Recall, F1-scores, and Accuracy of 79% each.

Boulouard, Z. et al.^34^ utilized an Arabic dataset proposed by^35^, comprising 15,050 comments collected from YouTube, including content in Iraqi, Gulf, and Egyptian dialects. The dataset was annotated with two class labels: Hateful and Inoffensive. Feature representation was performed using TF-IDF, and classifiers evaluated included LR, NB, RF, SVM, and LSTM networks. The LSTM model achieved the best performance, with Precision 92%, Recall 74%, F1-score 82%, and Accuracy 82%.

Muaad, A. Y. et al.^36^ employed four Arabic dialect datasets, originally proposed by^33,37^, all collected from Twitter, investigating misogyny and sarcasm classification under both binary and multiclass scenarios. The first dataset included 7,866 tweets labeled as misogyny or non-misogyny (binary), and the second contained 6,550 tweets labeled across multiple misogynistic categories (discounting, stereotyping and objectification, damping, threat of violence, derailing, dominance, sexual harassment, and non-misogyny; multiclass). The third dataset comprised 15,548 tweets labeled as sarcastic or non-sarcastic (binary), and the fourth included 15,370 tweets labeled as positive, negative, or neutral for sarcasm multiclassification. Feature representations included BOW, TF-IDF, and BERT-based embeddings. Classifiers evaluated included AraBERT, AraBERTv2, PAC, LR, RF, Linear SVC (LSVC), Decision Tree (DT), and KNN. AraBERTv2 achieved the best performance across all datasets: first dataset: F1-score 90%, Accuracy 91%; second dataset: Accuracy 82%; third dataset: F1-score 77%, Accuracy 88%; fourth dataset: F1-score 75%, Accuracy 77%.

Mohdeb, D. et al.^38^ developed five datasets composed of comments written in the Algerian dialect along with other regional and foreign languages, all collected from YouTube. The first dataset contained 4,681 comments in Algerian Arabic, Arabizi, French, and English; the second comprised 4,247 comments in Algerian Arabic, French, and English; the third included 4,681 comments in Algerian Arabic, inverted Arabizi, French, and English; the fourth consisted of 4,240 comments in Algerian Arabic and inverted Arabizi; and the fifth dataset had 3,806 comments entirely in Algerian Arabic. All datasets were annotated with five categories: Incitement (I), Hate (H), Refusal with Non-Hateful words (RNH), Sympathetic (S), and Comment (C). Several pre-trained language models were evaluated, including mBERT, XLM-R, AraBERT, and DziriBERT. mBERT achieved the best results on the first three datasets: first dataset: Precision 49%, Recall 47%, F1-score 47%, Accuracy 59%; second dataset: Precision 53%, Recall 47%, F1-score 46%, Accuracy 60%; third dataset: Precision 51%, Recall 46%, F1-score 46%, Accuracy 60%. AraBERT outperformed others on the fourth and fifth datasets: fourth dataset: Precision 62%, Recall 55%, F1-score 56%, Accuracy 67%; fifth dataset: Precision 59%, Recall 56%, F1-score 57%, Accuracy 68%.

Alhayan, F. et al.^39^ collected an Arabic dataset in the Saudi dialect, comprising 7,487 tweets from Twitter, annotated for binary classification into hateful and non-hateful categories. Feature representations included TF-IDF and Part-of-Speech (POS) tagging. Machine learning classifiers evaluated were SVM, RF, DT, and XGBoost. The RF classifier achieved the highest performance: with TF-IDF features: Precision 87%, Recall 84%, F1-score 84%, Accuracy 85%; with POS features: Precision 85%, Recall 83%, F1-score 83%, Accuracy 84%.

Tashtoush, Y. et al.^40^ collected an Arabic dataset in the Jordanian dialect, comprising 20,000 comments from YouTube, annotated for both binary and multi-class classification tasks. For binary classification, the labels were Bullying and Non-Bullying; for multi-class, the labels included General Bullying, Racism, Sexism, and Officials Bullying. Feature representation was performed using GloVe-Arabic embeddings. Four deep learning classifiers were evaluated: CNN, LSTM, Bi-LSTM, and a hybrid CNN-LSTM model. For the binary task, CNN and CNN-LSTM achieved the best performance, each with Precision 91.7%, Recall 92.1%, F1-score 91.9%, and Accuracy 91.9%. For the multi-class task, LSTM achieved 89.6% for Precision, Recall, and F1-score, and 89.5% for Accuracy, and Bi-LSTM achieved Precision 89.8%, Recall 89.5%, F1-score 89.6%, Accuracy 89.5%.

Awlla, K. M. et al.^41^ utilized a Central Kurdish dataset originally proposed by^42^, comprising 14,881 comments collected from Facebook, annotated with three sentiment classes: 9,133 positive, 3,837 negative, and 1,911 neutral. Feature representation employed BERT embeddings, and deep learning classifiers evaluated included BiLSTM, Multi-Layer Perceptron (MLP), and Fine-Tuned BERT. The Fine-Tuned BERT model achieved the highest performance across all classes, with an F1-score of 78% and Accuracy 75.37%. After removing neutral samples for binary classification (positive vs. negative), the Fine-Tuned BERT model achieved F1-score 87% and Accuracy 86.31%. These findings highlight the effectiveness of supervised learning methods for sentiment analysis in low-resource languages, aligning with the conclusions of^43^. However, as demonstrated in^44^’s work on Kurdish Named Entity Recognition (NER), substantial challenges persist in scaling transformer models to more nuanced NLP tasks in low-resource contexts, due to dialectal variability and the limited availability of annotated data.

Table 1 shows a summary of the related work in Arabic toxicity detection and illustrates each study: dataset creation, dataset size, collection source(s), class names, dialects, feature representations, best classifiers, and performance metric values in terms of precision (Pre), recall (Rec), F1-score (F1), and accuracy (Acc).

It is apparent that research on toxicity detection in Arabic remains limited, with several existing studies exhibiting low performance and lacking robust features and text representations. Moreover, instead of creating new datasets, most studies have relied on previously published datasets.

**Table 1:** Summary of related work on Arabic toxicity detection.

Study	Dataset creation	Dataset size	Source	Classes		Dialect	Feature representation		Best Classifier		Performance (%)			
											Pre	Rec	F1	Acc
Harbaoui, A. et al.^13^	USED	32,000	Aljazeera	Bully, Non-Bully		MSA and Different Dialects	Sentence Embeddings: SEG Module, PLMs		USE + LR		34	81	48	-
		12,896	Formspring.me			English			USE + XGB		36	75	49	-
		6,580	Impermium			English			RoBERTa (FT)		72	86	78	-
Lee, N. et al.^17^	USED	12,698	Twitter	Hate, Non-Hate		MSA and DA	Sentence Transformers: SBERT Embeddings		AraBERTv0.2-Twitter_base_		84	80	82	-
		40,429	NAVER news, and YouTube			Korean			KcELECTRA_base_−v2022		83	80	81	-
		150,000	Twitter, Reddit, and HateSites			English			BERTweet_base_		86	86	86	-
Mazari, A. C. and Kheddar, H.^21^	BUILT	14,150	Facebook, YouTube, Twitter	hate speech, offensive language, cyberbullying		Algerian	Word Embeddings (ML)	TF-IDF	ML Classifiers	MNB	-	-	73.6	66.3
										SGD	-	-	72.3	71.6
								Word2vec (Skip-Gram)		MNB	-	-	68.2	53.4
										SVC	-	-	63.4	55.7
								Word2vec (CBOW)		SGD	-	-	72.4	56.9
							Word Embeddings (DL)	FastText	DL Classifiers	Bi-GRU	-	-	75.8	73.6
Harrag, F. et al.^22^	USED	3,353	Twitter	target	Race, Gender, Religion, Ethnicity, Other	MSA and Arabic Dialects	Not Mentioned		AraBERT		78	81	78	81
Alrashidi, B. et al.^23^	USED	3,353	Twitter	directness	Direct, Indirect	MSA and Arabic Dialects	TF-IDF, AraVec, NLPaug library		MARBERT		68	65	65	67
				hostility	Abusive, Hateful, Offensive, Disrespectful, Fearful, Normal				MARBERT		45	46	44	46
				target	Origin, Gender, Religion, Disability, Other				CAMeLBERT		81	82	82	81
				group	Individual, Other, Women, Special needs, African descent				MTL (MARBERT + BiLSTM + CNN)		-	-	91	89
				annotator	Disgust, Shock, Anger, Sadness, Fear, Confusion, Indifference				CAMeL-BERT		56	57	56	57
Al-Hassan, A. and Al-Dossari, H.^26^	BUILT	11,000	Twitter	Religious, Racism, Sexism, General hate speech, Nonhate		Dialectical Arabic	Word Embeddings: TF-IDF, Keras Embedding		CNN + LTSM		72	75	73	-
Badri, N. et al.^27^	USED	1,101	Twitter	Normal, Hate and Abusive		Egyptian	Word Embeddings: AraVec, FastText, TF-IDF		CNN-BiGRU + FT and CNN-BiGRU + FT + AraVec		64	63	63	-
		6,477	Facebook, YouTube			Tunisian			Random forest		81	81	81	-
		9,200	Twitter			Lebanese			CNN + FT + AraVec		79	75	77	-
		23,033	Facebook, YouTube, Twitter			Egyptian, Tunisian, Lebanese			CNN-BiGRU + FT + AraVec		82	81	82	-
Husain, F. and Uzuner, O.^30^	USED	1,100	Twitter	Offensive, Not offensive		Egyptian	Not Mentioned		AraBERT		78	78	78	78
		6,024				Tunisian					79	80	80	81
		5,846				Levantine					86	87	87	87
		10,000				Multidialect					86	87	87	87
Masadeh, M. et al.^32^	USED	6,164	Twitter	Hate, Not Hate		Dialectal Arabic	BOW, TF-IDF, Word2Vec, BERT		Ara-BERT		79	79	79	79
Boulouard, Z. et al.^34^	USED	15,050	YouTube	Hateful, inoffensive		Iraqi, Gulf, and Egyptian	TF-IDF		LSTM		92	74	82	82
Muaad, A. Y. et al.^36^	USED	7,866	Twitter	Binary Class	Nonmisogyny, Misogyny	Dialectal Arabic	BOW, TF-IDF, BERT		AraBERTv2		-	-	90	91
		6,550		Multi-Class	Discredit, Stereo typing and objectification, Damning, Threat of violence, Derailing, Dominance, Sexual harassment, Nonmisogyny				AraBERTv2		-	-	-	82
		15,548		Binary Class	Nonsarcastic, Sarcastic				AraBERTv2		-	-	77	88
		15,370		Multi-Class	Positive, Negative, Neutral				AraBERTv2		-	-	75	77
Mohdeb, D. et al.^38^	BUILT	4,681	YouTube	Incitement (I), Hate (H), Refusing with nonhateful words (RNH), Sympathetic (S), Comment (C)		Algerian Arabic, Algerian, Arabizi, French and English	Not Mentioned		mBERT		49	47	47	59
		4,247				Algerian Arabic, French, and English			mBERT		53	47	46	60
		4,681				Algerian Arabic, Converted Algerian Arabizi, French and English			mBERT		51	46	46	60
		4,240				Algerian Arabic, Converted Algerian Arabizi			AraBERT		62	55	56	67
		3,806				Algerian Arabic			AraBERT		59	56	57	68
Alhayan, F. et al.^39^	BUILT	7,487	Twitter	Hateful, Non-hateful		Arabic (Saudi)	TF-IDF		Random Forest		87	84	84	85
							POS Tagging				85	83	83	84
Tashtoush, Y. et al.^40^	BUILT	20,000	YouTube	Binary Class	Bullying, Nonbullying	Arabic (Jordanian)	GloVe-Arabic		CNN and CNN-LSTM		91.7	92.1	91.9	91.9
				Multi-Class	General Bullying, Racism, Sexism, Officials Bullying				LSTM		89.6	89.6	89.6	89.5
									Bi-LSTM		89.8	89.5	89.6	89.5
Awlla, K. M. et al.^41^	USED	14,881	Facebook	Positive, Negative, Neutral		Central Kurdish	BERT		Fine-Tuned BERT		-	-	78	75.37
				Positive, Negative									87	86.31

Notably, the relationships between the previous studies on OSNs and our study were examined and compared. While most prior research relied on datasets developed by others, we constructed a new, standard dataset for toxicity and abuse detection through manual annotation. Based on our pertinent research, only a limited number of studies have addressed toxicity detection in the Arabic language. Therefore, our considerable work has investigated toxicity detection in the Arabic language. There is a diversity among the Arabic dialects that is emphasized in this study rather than the previous ones. Our corpus, consisting of nearly 50,000 balanced Arabic tweets, is the largest among most previous Arabic studies. We applied sixteen different machine learning models, the FastText model (in its default form), and seven transfer learning models to detect toxicity in Arabic content on OSNs, particularly on Twitter. Our study achieved the highest performance in terms of F1-score and accuracy metrics when compared with those of previous studies.

## Background

In this study, we used various word embedding techniques along with many algorithms, including transformers, by using BERT models specialized in the Arabic language. The purpose of using this all-encompassing method was to validate our suggested dataset.

### Bag of words (BoW)

It is a method of numerically representing text that transforms text into a numerical digital vector depending on how frequently each token appears in the text documents. When BoW is used, there is a loss of semantic and syntactic information and order between words^45,46^.

### Term frequency – inverse document frequency (TF-IDF)

The term frequency (TF) is similar to that of the BoW technique, but the TF depends on the repetition of the term in the provided document, whereas the BoW depends on its occurrence. Therefore, we can discuss the TF as it is the number of times that a specific term appears in the document^47,48^. Equation (1) expresses all about TF.

1 $$\:TF=\:\frac{Number\:of\:times\:the\:term\:appears\:in\:the\:document}{Total\:EquationNumber\:of\:terms\:in\:the\:document}$$

The inverse document frequency (IDF) is the total number of documents that include this specific term. It is expressed in Eq. 2.

2 $$\:IDF=\:{\text{Log}}_{10}(\:\frac{Total\:EquationNumber\:of\:the\:documents\:in\:the\:corpus}{Number\:of\:documents\:in\:the\:corpus\:contains\:the\:term}\:)$$

Term frequency–Inverse document frequency (TF-IDF) is calculated by multiplying TF by IDF. Equation 3 expresses that.

3 $$\:TF-IDF=TF*IDF$$

### FastText

It is a library developed by Facebook’s AI research team^49^ for very reliable word embedding and text classification learning techniques. This FastText model selects the embeddings from the character n-grams of the training words. The model enables the development of supervised and unsupervised learning algorithms, but here, we focused on supervised learning algorithms for the acquisition of word vector representations. This is formalized in Eq. (4).

4 $$\:-\frac{1}{N}\sum\:_{n=1}^{N}{y}_{n}\:\text{l}\text{o}\text{g}\left(f\right(BA{x}_{n}\left)\right)$$

where $\:{x}_{n}$ is the normalized bag of features of the n^th^ document, $\:{y}_{n}$ is the label of the n^th^ document, A and B are its weight matrices, $\:f$ is the SoftMax function, and $\:N$ is the total number of documents.

### Transformers

**Fig. 1:**
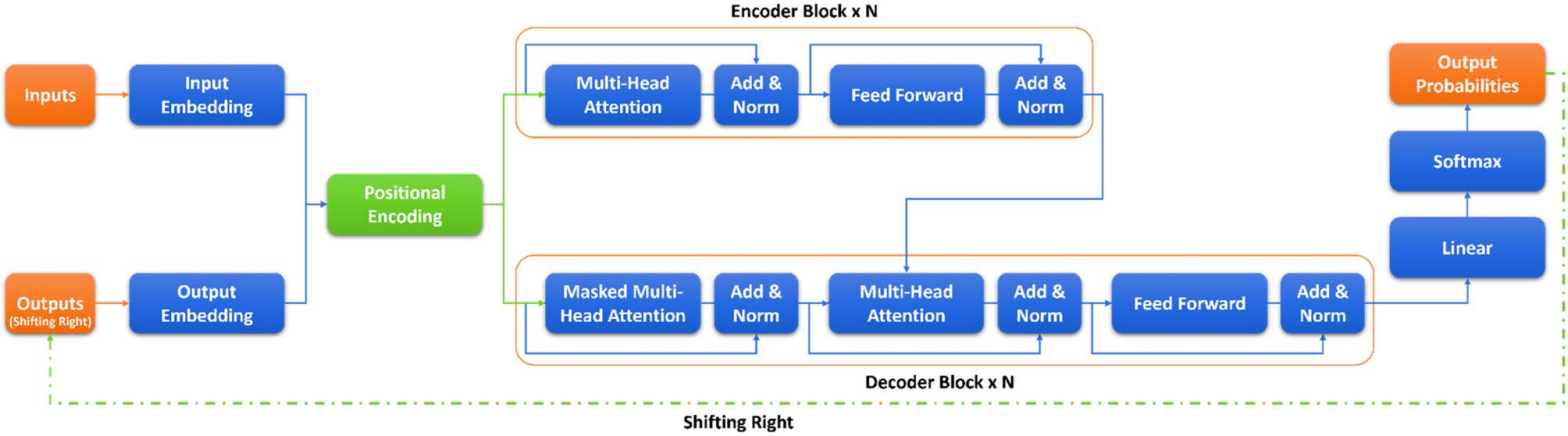
Architecture of the transformer of the NLP.

Transformers, also known as “Attention is All You Need”, introduced by^50^, have revolutionized the fields of natural language processing (NLP) and machine learning. These models employ a mechanism known as self-attention, allowing them to capture long-range dependencies and relationships within input sequences effectively. Figure 1 shows the transformer architecture in NLP. Their architecture, consisting of multiple self-attention layers and feedforward neural networks, has led to remarkable performance in various NLP tasks^51^. The transformer architecture has since become the backbone of numerous state-of-the-art models, including BERT^51^. Its ability to model contextual information and handle sequential data efficiently has made transformers a cornerstone in modern deep learning research, significantly impacting diverse applications across language translation, text generation, and beyond. The self-attention mechanism in transformers involves calculating attention scores for each word/token in a sequence. The attention score for a query ​and a key can be computed via the scaled dot-product attention function in Eq. 5.

5 $$\:Attention\left(Q,\:K,\:V\right)=softmax\left(\frac{Q{K}^{T}}{\sqrt{{d}_{k}}}\right)V$$

where Query (Q), Key (K), Value (V), Time (T), and d_k_ represent the dimensionality of the keys and queries. Figure 1 shows the architecture of the transformers.

#### Bidirectional encoder representations from transformers (BERT)

**Fig. 2:**
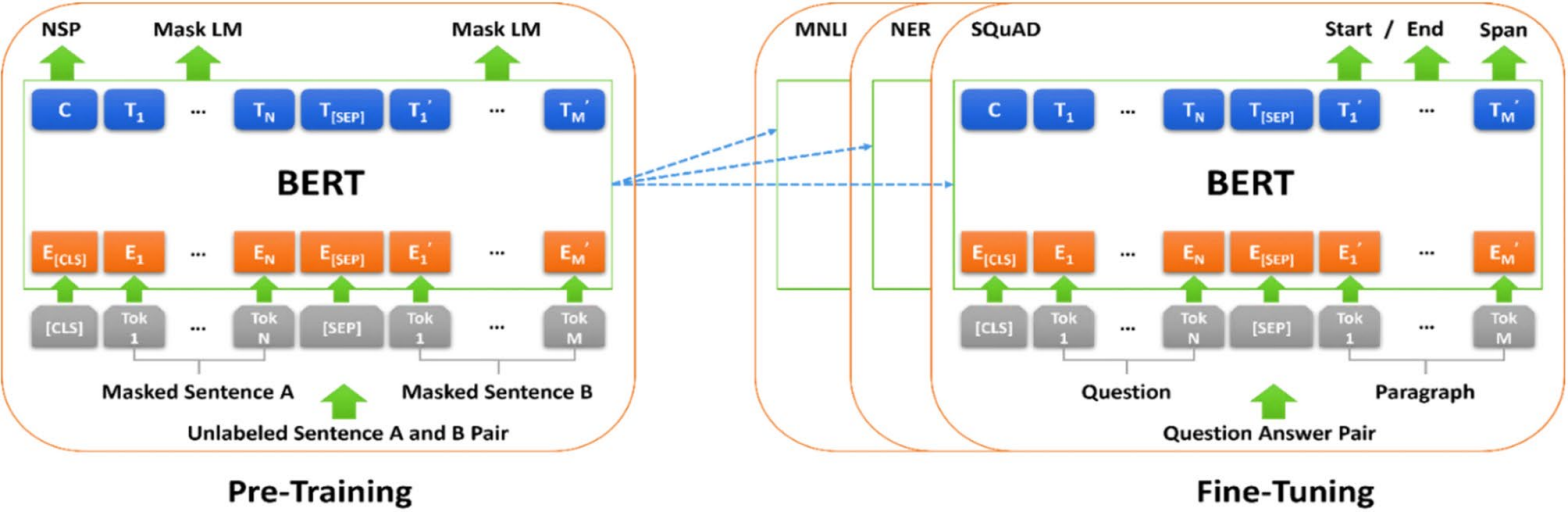
BERT architecture in terms of the pretraining and fine-tuning phases.

The Transformer neural network’s parallel attention layers serve as a fundamental component in BERT, a contextualized word representation model, replacing sequential recurrence in the multilayer bidirectional transformer encoder^32^. Introduced by^51^, BERT operates within a framework comprising two primary phases: pretraining and fine-tuning. During the pretraining phase, the model is constructed using unlabeled data obtained from numerous pretraining tasks. In the fine-tuning phase, the pre-trained parameters are subsequently employed to initialize the model, and through the utilization of labeled data, all the parameters are further refined and optimized. This approach allows BERT to leverage the vast amounts of unlabeled data available during pretraining while also fine-tuning its parameters to specific tasks via labeled data, ultimately enhancing its performance and adaptability. Figure 2 shows the architecture of BERT in terms of the pretraining and fine-tuning phases. The algorithms that are based on BERT for Arabic language models include AraBERT, AraBERTv02, ARBERT, MARBERT, MARBERTv2, multilingual BERT, and Arabic-BERT^52^.

In the next section, we delve into a discussion of the most prevalent BERT models specifically tailored for processing Arabic text.

##### AraBERT

AraBERT is a large step forward in Arabic natural language processing (NLP). It is a BERT-based^51^ model that has been designed to solve the difficulties and constraints of Arabic language processing, such as morphological complexities and dialectal variances. The architecture of AraBERT focuses on exploiting large-scale pretraining on various Arabic text sources to properly collect contextual information, hence improving performance across many NLP tasks^53^.

##### AraBERTv02

AraBERTv02 is a developed version of the original AraBERT model. This version of AraBERT is likely to include innovations in pretraining approaches, architecture changes, or fine-tuning tactics. AraBERTv02 attempts to improve overall performance by refining the model’s capacity to grasp and create text in Arabic, adjusting to subtle linguistic aspects, and optimizing Arabic language processing tasks^53^.

##### ARBERT

ARBERT (also known as ARabic BERT) is a famous pre-trained language model that was created exclusively for Arabic text processing. It strives to increase Arabic NLP skills by taking into account contextual subtleties and language complexities. ARBERT, a BERT-based^51^ model, seeks to deliver stable representations and improve performance on several downstream NLP tasks in Arabic^54^.

##### MARBERT

MARBERT is a multidialectal and multilingual Arabic BERT model, also known as multilingual ARabic BERT. It includes many dialects and variants of the Arabic language to capture distinct linguistic subtleties and improve Arabic NLP skills across various Arabic-speaking regions. The architecture of MARBERT accommodates the intricacies of Arabic language structures and variances, resulting in increased performance in a variety of NLP applications^54^.

##### MARBERTv2

The term MARBERTv2 refers to an upgraded or enhanced version of the MARBERT model. To improve its performance on Arabic language-related tasks, it is likely to include innovations in pretraining procedures, model architecture, or fine-tuning strategies. This version is planned to improve on MARBERT’s strengths, perhaps by providing stronger representations and better adaptation to varied Arabic language variances^54^.

##### Multilingual BERT

Multilingual BERT is a BERT-based^51^ approach that supports a variety of languages, including Arabic. It combines multilingual capabilities by simultaneously learning representations from many languages, offering a wide basis for NLP tasks in a variety of linguistic situations. While not exclusively focused on Arabic, its multilingual nature enables the use of Arabic-language data with other languages for full NLP applications^55^.

##### Arabic-BERT

Arabic-BERT is a pretraining model for the Arabic language that aims to improve Arabic text comprehension and generation. It is designed to accommodate the intricacies and complexity of the Arabic language, allowing for improved performance in different NLP tasks suited for Arabic text^52^.

## Methodology

**Fig. 3:**
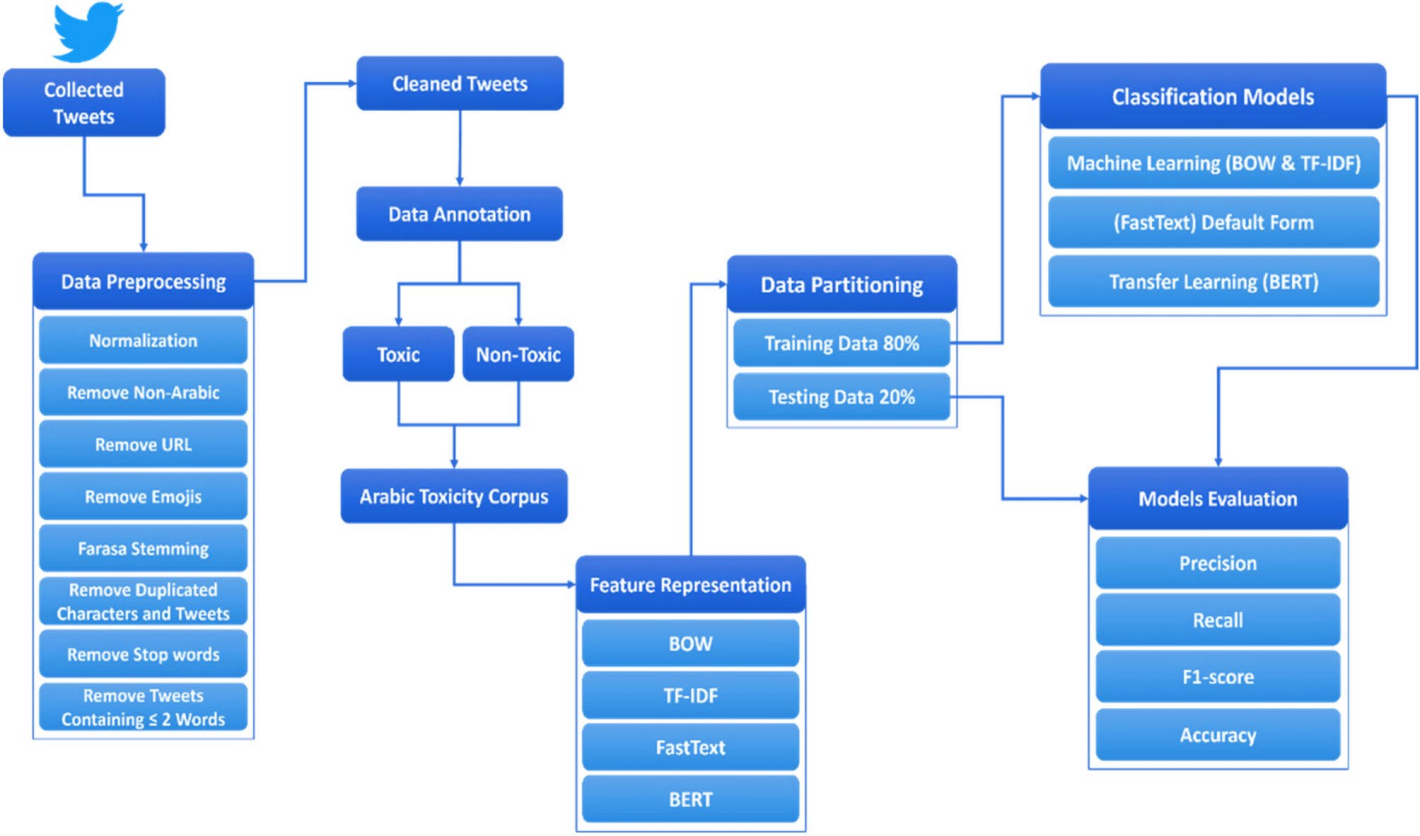
The proposed technique for Arabic toxicity detection.

The proposed technique for Arabic toxicity detection is shown in Fig. 3. It includes the following major steps: collected tweets, data preprocessing, text representation, the building of classification models, and model evaluation. The pseudocode of this proposed technique is listed in Algorithm 1.

The following pseudocode shows the methodology’s steps:

**Algorithm 1:**
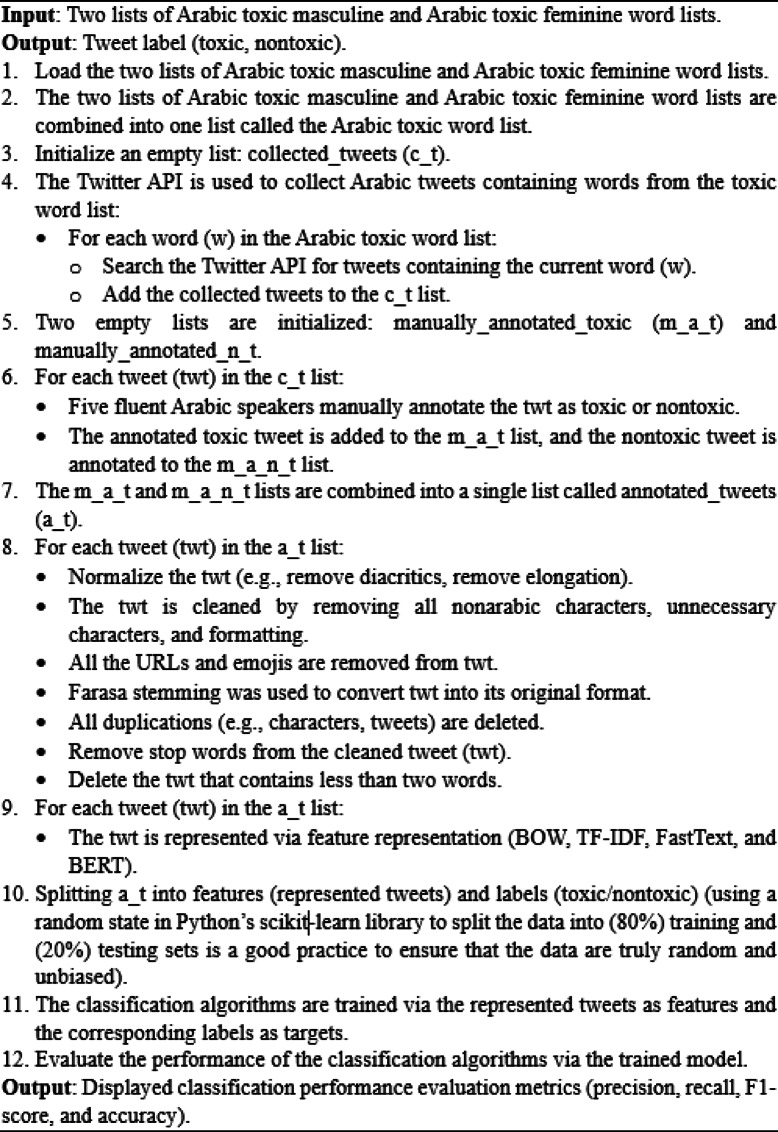
Methodology steps: Dataset construction, preprocessing, feature representation, classification, and evaluation.

### Dataset

**Fig. 4:**
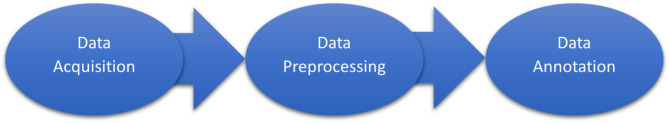
Dataset development phases.

We built our dataset by using the concept of dataset development phases in Fig. 4. The dataset development phase consists of three steps: data acquisition, data preprocessing, and data annotation. Each of these steps is related to the other, as in the data acquisition phase, we collected our dataset; in the data preprocessing phase, we cleaned and filtered our dataset; and in the final data annotation phase, we labeled our dataset. We used these development phases to make our dataset ready for use.

### Data acquisition: collection of tweets

In this study, we used Twitter API^56^ with a cursor by using the Tweepy Python library^57^ and our Twitter API credentials for authentication to collect the dataset from Twitter. A search was performed for each word in the keyword list, as shown in Table 2, and all the retrieved tweets were collected and saved in the dataset. The list of hate speech categories introduced by^58^ presents the tenth fundamental meaning in the vocabulary for the abuse in the Arabic language. We have added many more examples. In addition, we divided the examples into two lists of Arabic masculine words and Arabic feminine words that attract and trigger toxic content. We collected approximately 80,000 Arabic tweets. Table 2 contains the Arabic masculine and feminine words that have been used in the search query parameter categorized into toxicity characteristics and their English translation.

**Table 2:** Arabic masculine and feminine words categorized according to their toxic characteristics and their translation into English.

Toxic speech meaning	Arabic masculine words	Arabic feminine words	English translation
Low	حقير – وضيع – منبوذ	حقيرة – وضيعة – منبوذة	Despicable – Mean – Outcast
Dirty	نجس – معفن – وسخ – قذر – مقرف	نجسة – معفنة – وسخه – قذرة – مقرفة	Impure – Moldy – Dirty – Filthy – Disgusting
Double faced	منافق – عميل – خاين	منافقة – عميلة – خاينة	Hypocrite – Agent – Traitor
Irrational & unreasonable	مجنون – متخلف – أهبل – اهطل – معتوه	مجنونة – متخلفة – هبله – هطله – معتوهه	Crazy – Subnormal – Asshole – Stupid – Imbecile
Sick	مريض – يشفي الكلاب ويضرك – تعبان فدماغه – اقرع – أطرش	مريضة – يشفي الكلاب ويضرها – تعبانة فدماغها – طرشه	Sick – God heals the Dogs and Harm you – Confused – Bald – Deaf
Immoral & irreligious	كافر – مرتد – عاهر – شرموط – ديوث – ملحد – عرص – معرص – متناك – خول – بضان – طيز	كافرة – مرتدة – عاهره – شرموطة – ديوثه – ملحدة – معرصه – متناكة	Infidel – Apostate – Prostitute – Bitch – Cuckold – Atheist – Pimp – Disobedient – Sexual Intercourse – Libidinous – Testicles – Ass
Shameless	وقح – سافل – منحط – مهزق – أحمق – علء	وقحه – سافله – منحطة – مهزقه – حمقاء – قحبة	Shameless – Bastard – Degenerate – Licentious – Foolish – Worthless – Whore
Ethnic labels	فلاح - صعيدي – يهودي	فلاحة – صعيدية – يهودية	A Farmer – From Upper Egypt – A Jew
Animal	خنزير – حمار – كلب – خروف – تيس – براس كلبة – تور	خنزيرة – حمارة – كلبة – خروفة – لبوة – نعجة – بقرة – جاموسة (جموسة) – حشرة	Pig – Donkey – Dog – Sheep – Goat – Dog Head – Bull – Lioness – Ewe – Cow – Buffalo – Insect
Jobs	عربجي – زبال – بواب – شحات	عربجية – زبالة – بوابة – شحاتة	Junk Seller – Caterer – Scavenger – Concierge

### Data preprocessing

When we collect data from Twitter, these gathered data may contain text that is not useful for classification, such as non-Arabic letters, URLs, or emojis. This can negatively affect the performance of the classification models. Therefore, the data must be cleaned and filtered before they can be used for classification. To be more helpful for text representation and learning tasks, tweets must be preprocessed. We used various techniques to prepare the text for further processing. As shown in Fig. 3, we filter these preprocessing technique steps on our dataset. The first stage is normalization^59,60^, which is a crucial stage in transforming the text into a uniform format so that the computer can simply read and understand the content. During this process, all letters are normalized into their original form. For example, with respect to normalization, the letters in Arabic “أ”, “إ”, and “آ” changed to “ا”; the letter “ة” changed to “ه”; and the letters “ى” and “ئ” changed to “ي”. Deleting all “Tashkeel” Arabic diacritics, such as “fatha”, “damma”, “kasra”, and “tashdid”; and removing the elongation in Arabic text, such as “ســــــــــــــــام” (“toxxxic”), changed to “سام” (“toxic”). Then, non-Arabic removal was performed during this stage, and anything that was not Arabic (such as characters, numbers, and symbols) was removed. After that, we proceed with the URL removal and emoji removal steps, in which all links and emotions are removed. Then, a critical stage called the Farasa stemming^61^ stage was performed, which is an instance of the Arabic NLP Toolkit, which converts all words from any format into their original uniform format by removing suffixes, infixes, and prefixes of all words. For example, words in the Arabic language (“كلب”, “كلبة”, “ياكلب”, “كلاب”). “كلبة” has the prefix “ة” at the end, “ياكلب” has the suffix “يا” at the beginning, and “كلاب” has the infix “ا” in the middle. Using Farasa stemming, all of the phrases are compiled under the same keyword, “كلب” (“dog”), which is the root of all the above words. The Duplicated Characters and Tweets removal process then begins. Any repeated characters are removed (e.g., (سلااااااااااااام), which means that “Byeeeee” is changed to (سلام), which means “Bye”). Any duplicated tweets are deleted except for one. We then perform an important stage called the stop word removal stage, the main purpose of which is to eliminate words that have no value and are already present in many texts, which prevents the computer from understanding anything in the process of data analysis. In this study, we built a list of stop words called swds^62^ that contained all words that could be known as Prepositions (such as لكن (but),عن (about), على (on), في (in), مع (with), إلى (to), من (from), and أو (or)) to remove all the stop words from all the text in the dataset automatically. Finally, we removed tweets containing ≤ 2 words, and during this final stage, all tweets that contained words less than or equal to 2 words were deleted.

### Data annotation

**Fig. 5:**
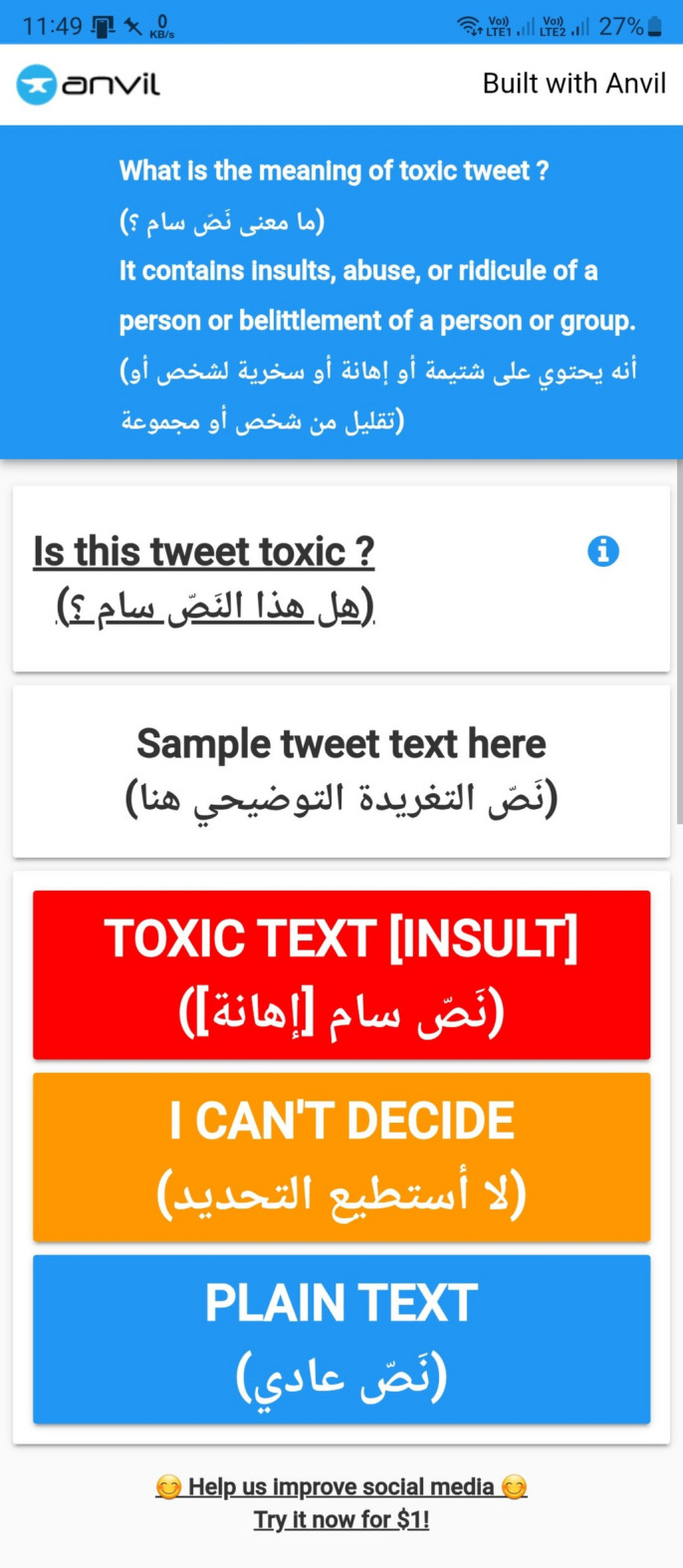
Annotation interface screenshot on a smartphone.

**Fig. 6:**
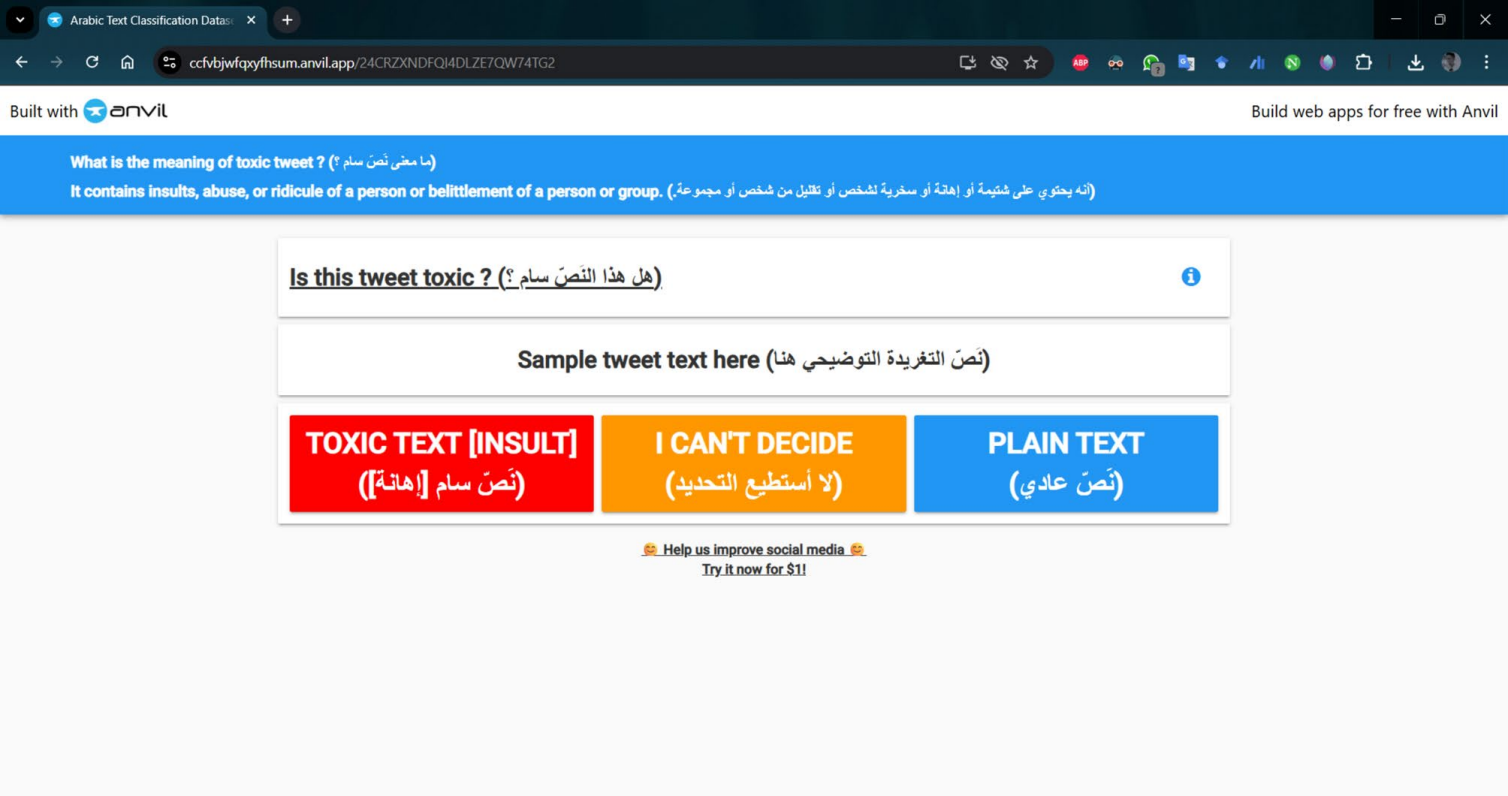
Annotation interface screenshot on a PC.

Five fluent and native Arabic speakers, representing diverse cultures, ages, genders, occupations, and well-educated backgrounds, manually labeled our dataset as Toxic or Non-Toxic. Each annotator labeled a disjoint subset of approximately 9,924 tweets, so that in total the five annotators covered the entire dataset of 49,620 tweets. To ensure consistent and reliable annotations, we provided clear criteria and detailed annotation guidelines, conducted a training session with example cases, and developed a custom-built web-based annotation platform in Python using the Anvil framework^63^, which enforced a uniform workflow for all annotators. In addition, periodic calibration meetings were held to resolve ambiguous cases and refine the guidelines. As illustrated in Figs. 5 and 6, annotators were presented with a prompt on the interface asking: “ما معنى نَصّ سام؟” (“What does a toxic text mean?”), followed by the clarification: “إنه يحتوي على شتيمة أو إهانة أو سخرية لشخص أو تقليل من شخص أو مجموعة.” (“It contains insults, offensive language, mockery of a person, or belittling of a person or group.”). These instructions were adapted from the United Nations definition of hate speech, which describes it as “*any kind of communication in speech*,* writing or behavior*,* that attacks or uses pejorative or discriminatory language with reference to a person or a group on the basis of who they are*,* in other words*,* based on their religion*,* ethnicity*,* nationality*,* race*,* color*,* descent*,* gender or other identity factor*”^64^. Building on this, our operational definition of toxicity encompassed hate speech, insults, threats, profanity, cyberbullying, and discriminatory expressions. Non-toxic tweets, by contrast, were those that lacked harmful, abusive, or degrading content, even if they expressed criticism or strong opinion. However, because the offensiveness of Arabic expressions is often shaped by dialectal and cultural context, annotators were instructed to evaluate both literal meanings and dialectical and contextual implications. For instance, the word “كلب” (“dog”) is a severe insult in Egyptian and Libyan dialects despite its neutral literal meaning, while “شرموطة” (“prostitute”) is a strong slur in Levantine and Egyptian usage. Similarly, “فلاح” (“farmer”) is typically neutral but may carry derogatory connotations in some Egyptian urban contexts. Other examples include “مطي” (“donkey”) in Iraqi Arabic, used as a degrading insult; “ديوث” (“cuckold”) in Saudi dialects, targeting morality and honor; “أطرش” (“deaf”) in Yemeni usage, employed mockingly; “خنزير” (“pig”) in Qatari Arabic, regarded as extremely degrading; and religiously loaded terms in MSA such as “كافر” (“infidel”) or “مرتد” (“apostate”), which attack identity and faith. These examples demonstrate how annotators relied on both linguistic nuance and cultural knowledge to ensure that toxicity labels reflect real-world Arabic usage rather than dictionary definitions alone. Figures 5 and 6 show the screenshots of the annotation interface as viewed on a smartphone and PC, respectively.

### Arabic toxicity corpus

**Fig. 7:**
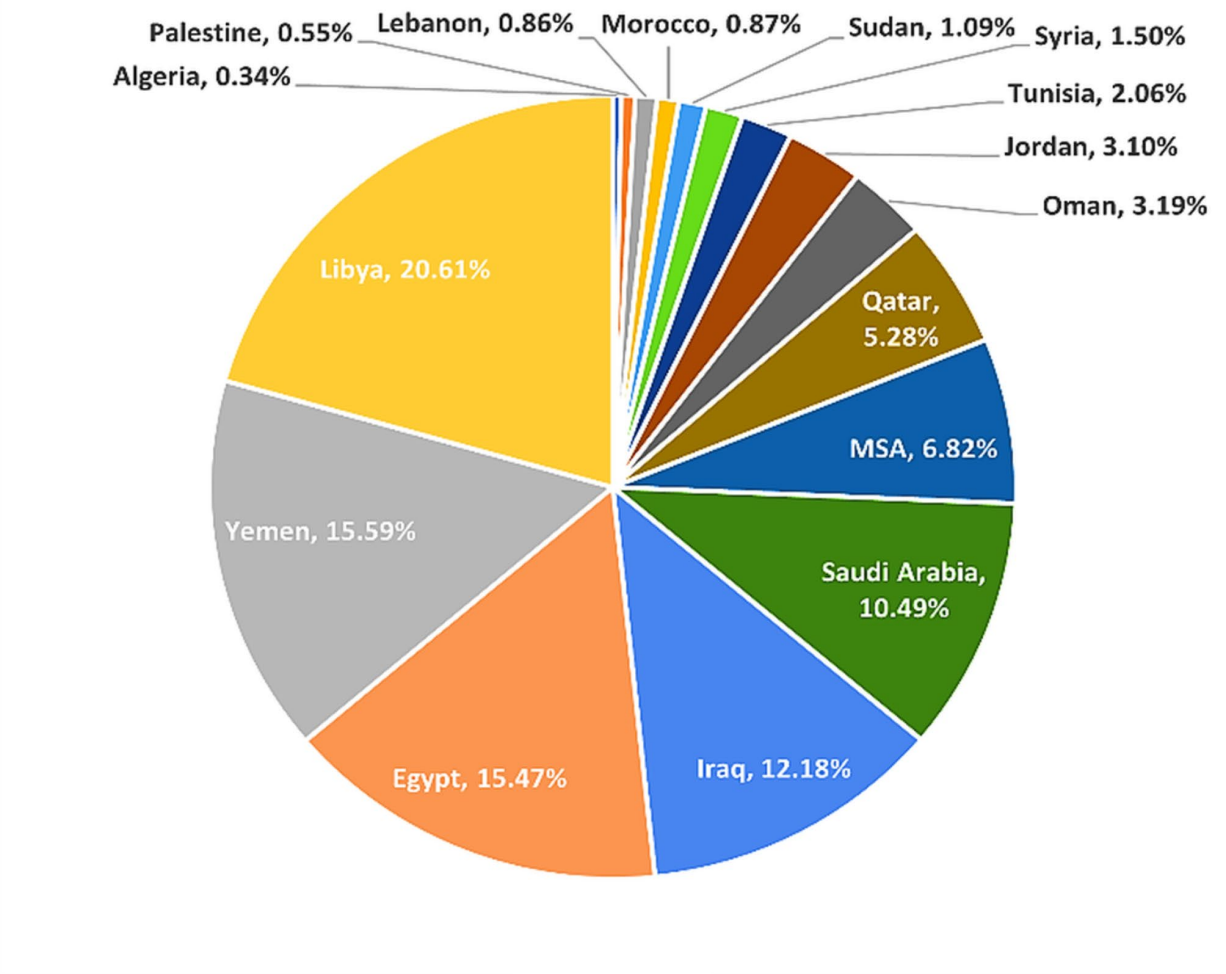
Arabic region dialect percentages in our corpus.

**Fig. 8:**
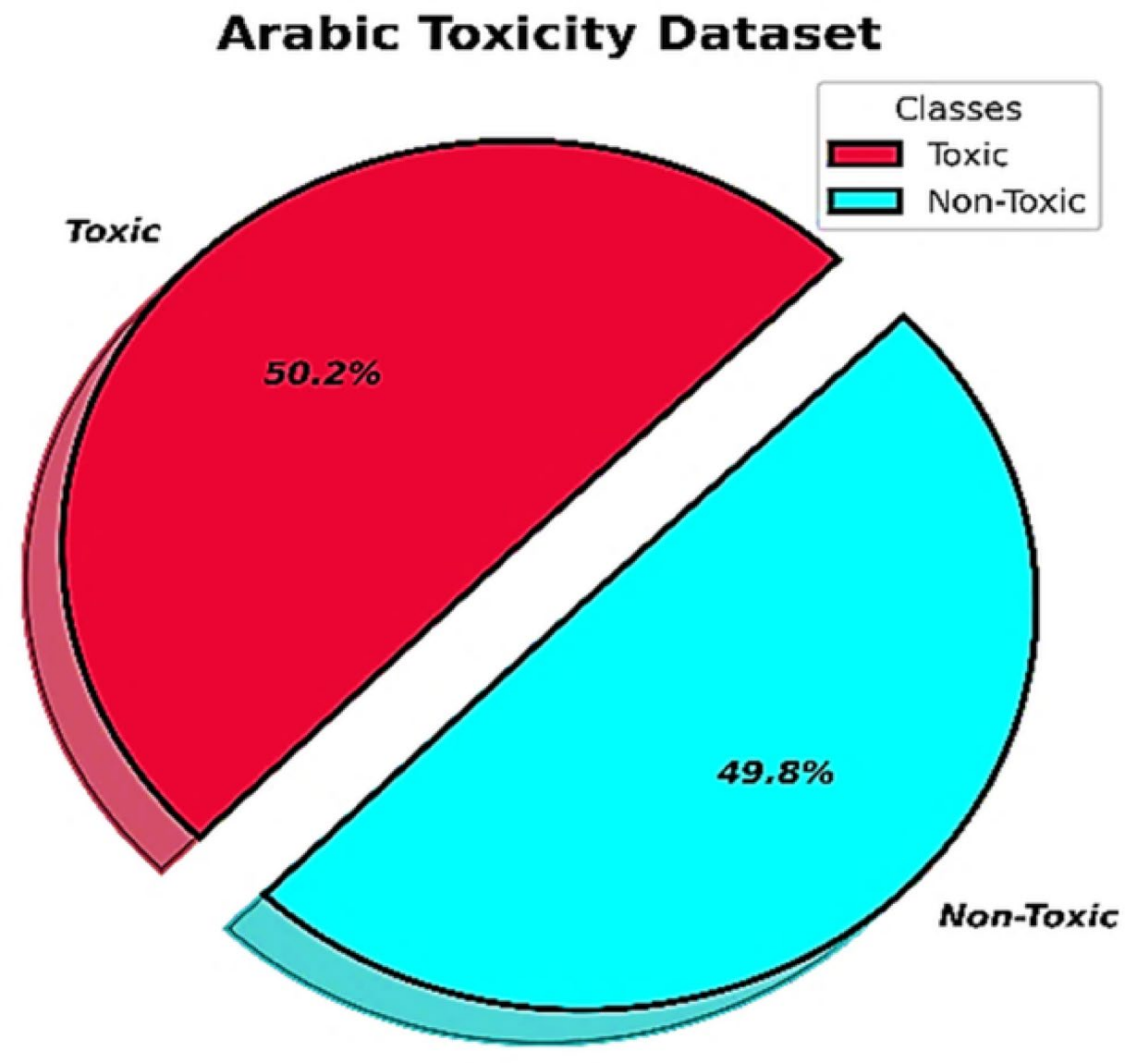
The percentage of tweets in each class.

In this section, we present some statistics, summarizations, and updates of our Arabic toxicity corpus after completing the three dataset development phases, namely, data acquisition, data preprocessing, and data annotation. After the data preprocessing and data annotation phases, we obtained a balanced corpus of 49,620 labeled tweets out of 80,000 collected tweets. There are 24,713 tweets from the toxic class and 24,907 tweets from the nontoxic class. We used the CAMeL API^65^ as an Arabic dialect identification tool for each tweet in the dataset. Our dataset contains a variety of various Arabic dialects from different countries. Figure 7 shows the Arabic region dialect percentages in our corpus. Figure 8 shows the percentage of tweets in each class.

As shown in Fig. 7, Libya, Yemen, Egypt, Iraq, Saudi Arabia, MSA, and Qatar are the most Arabic region dialects used in the dataset. However, the Libyan, Yemeni, and Egyptian dialects are the largest dialects focused on in the dataset.

### Feature representation

**Fig. 9:**
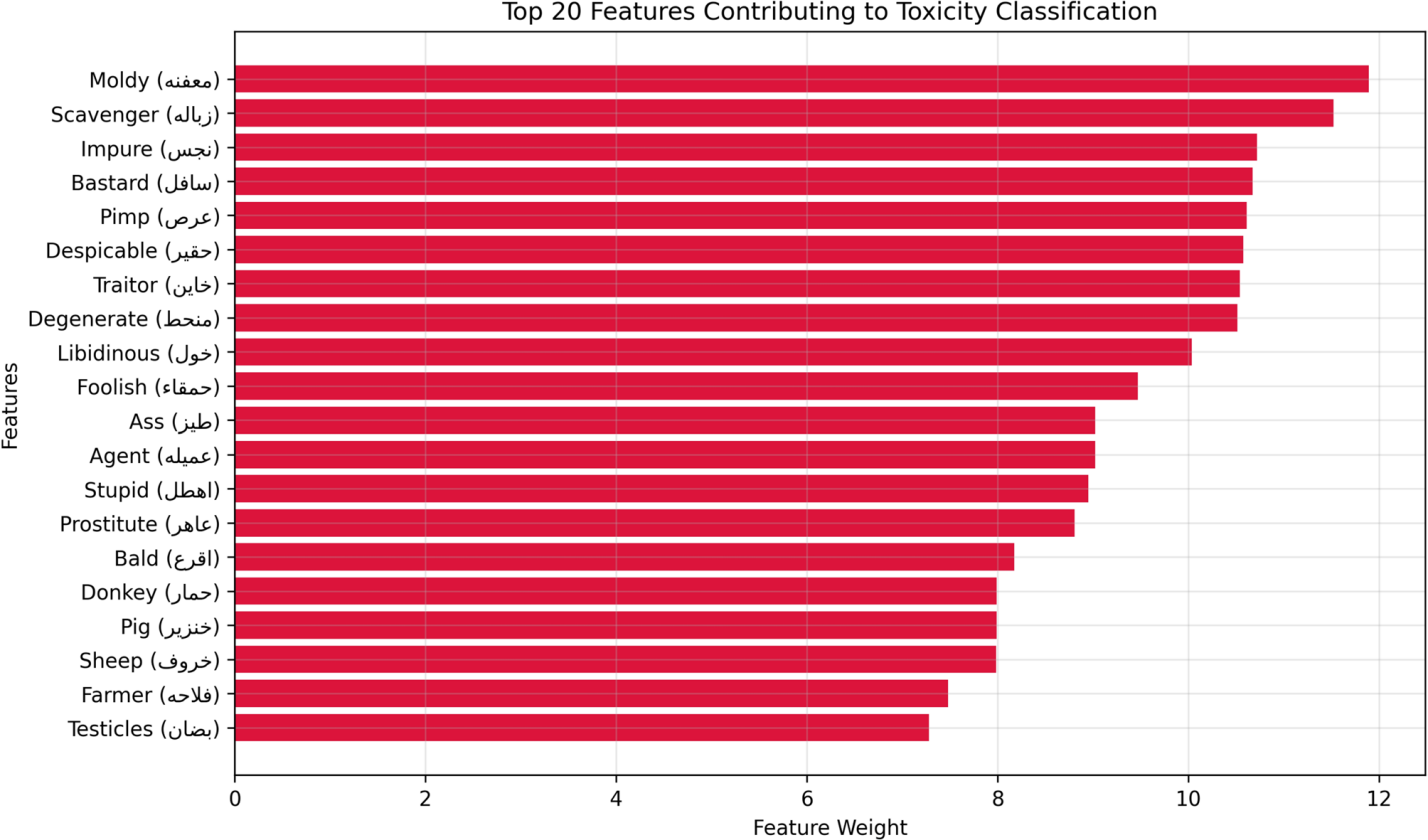
Top 20 features contributing to toxicity classification.

After building and preparing our dataset, our corpus is ready for representation purposes. The extraction of features from raw data is the most important phase for the computer, as it cannot manipulate raw data as string data. Therefore, several methods exist for transforming textual data (such as words, sentences, paragraphs, and documents) into numerical digital vector data, which machine learning algorithms can understand. We investigated the most popular word embedding techniques, including bag-of-words (BoW), term frequency-inverse document frequency (TF-IDF), FastText, and bidirectional encoder representations from transformers (BERT)^66^. The BoW and TF-IDF representations capture surface-level textual features, such as the presence or frequency of toxic terms, slurs, or offensive language, providing useful signals for detecting explicit toxicity. FastText captures subword and morphological patterns, enabling the detection of offensive variations (e.g., misspelled or stylized insults). BERT-based embedding captures deeper contextual and semantic meaning, which helps identify implicit or nuanced toxic expressions, such as sarcasm, dialectal threats, or indirect abuse^66^. These feature types allowed our models to detect a range of toxicity types, from explicit hate speech to subtle and context-dependent abuse, as illustrated by the top 20 toxic features presented in Fig. 9.

### Data partitioning

After we finish representing the text of our corpus in several ways, we need to split our corpus to prepare it for text classification. We used the training test split function to split the dataset into two parts: training data and testing data. The training data contains a larger amount of data from the dataset, and it is used for training the model. On the other hand, the testing data is used to test the model’s performance. Since there is no flawless division ratio, several ratios have been employed in the literature for Arabic text classification^59^. In our study, we partitioned our dataset into 80% training data and 20% testing data.

### Building of classification models (Learning)

Several types of algorithms were found to be more appropriate for various learning tasks. Making the appropriate choice is essential to the success of the text classification performance process. Many traditional machine learning and transfer learning techniques are available for natural language processing (NLP) applications such as text classification, but every technique has its limitations. In this study, we utilized the Python language with the Scikit Learn Library^67^ as in the supervised learning model for the classification phase^43^. We selected sixteen of the most widely recognized machine learning algorithms for Arabic text classification, such as Multinomial NB, Complement NB, Bernoulli NB, SVC, NuSVC, LinearSVC, Logistic Regression, Decision Tree, Random Forest, Neural Network, Gradient Boosting, Ridge, SGD, Perceptron, Nearest Centroid, and XGBoost^67^, , and FastText in its default form as supervised text classification^49^. Furthermore, we selected seven widely recognized transfer learning algorithms for Arabic text classification, including AraBERT, AraBERTv02, ARBERT, MARBERT, MARBERTv2, multilingual BERT, and Arabic-BERT^52^. These algorithms are all based on BERT^51^. We used bag of words (BoW) and term frequency–inverse document frequency (TF-IDF) as a text representation with the sixteen machine learning algorithms, selected FastText as a text representation as its default form for supervised text classification and utilized bidirectional encoder representations from transformers (BERT) as a text representation with the seven transfer learning algorithms. Our experimentation involved identifying the best matches to achieve optimal performance for most classifiers via our dataset.

### Evaluation metrics

In this study, we used four well-known measurement techniques: precision (Pre) (Eq. 6), recall (Rec) (which is known as sensitivity) (Eq. 7), F1-score (F1) (Eq. 8), and accuracy (Acc) (Eq. 9). We utilized them as the evaluation performance metrics for the classification algorithms^68^. The performance metric formulas are defined as follows:

6 $$\:Precision=\frac{TP}{TP+FP}$$

7 $$\:Recall/Sensitivity=\frac{TP}{TP+FN}$$

8 $$\:F1-Score=2\:\times\:\:\frac{Precision\times\:Recall}{Precision+Recall}$$

9 $$\:Accuracy=\frac{TP+TN}{TP+TN+FP+FN}$$

where TP, TN, FN, and FP refer to true positive, true negative, false negative, and false positive, respectively. In these formulas, true positive (TP) refers to the number of correctly predicted toxic tweets. True negative (TN) is the number of correctly predicted nontoxic tweets. False positive (FP) is the number of non-toxic tweets incorrectly predicted as toxic. A false negative (FN) is the number of toxic tweets incorrectly predicted to be nontoxic. These values are typically organized in a confusion matrix, which provides a clear summary of the classification model’s performance by showing the counts of actual versus predicted labels across the different classes.

## Results and discussions

A technique was built to address the toxicity and harmful content of OSNs, with a focus on Arabic toxic tweet classification. For this experimentation, we conducted several experiments in a Kaggle environment to test the proposed strategies. We selected several ML and TL algorithms that were used with various feature representation methods. We selected sixteen machine learning algorithms, namely, Multinomial NB, Complement NB, Bernoulli NB, SVC, NuSVC, LinearSVC, Logistic Regression, Decision Tree, Random Forest, Neural Network, Gradient Boosting, Ridge, SGD, Perceptron, Nearest Centroid, and XGBoost algorithms^67^, and applied them with BOW and TF-IDF feature representations. We used FastText as its default form for text representation and classification^49^. Seven transfer learning algorithms were used, namely, AraBERT, AraBERTv02, ARBERT, MARBERT, MARBERTv2, multilingual BERT, and Arabic-BERT^52^, and they were applied with BERT representation. We review our results in terms of precision, recall, F1-score, and accuracy. However, we focus on the F1-score and accuracy metric evaluation for model comparison, as the F1-score is the harmonic mean of precision and recall and provides a balance between them^69^. Accuracy measures the overall correctness of the models across all classes and represents the ratio of correctly predicted observations (both true positives and true negatives) to the total number of observations^70^. In this section, four feature representation methods are used to assess the effectiveness and performance of this study. We clarify and discuss these experimental results.

### BOW representation

**Fig. 10:**
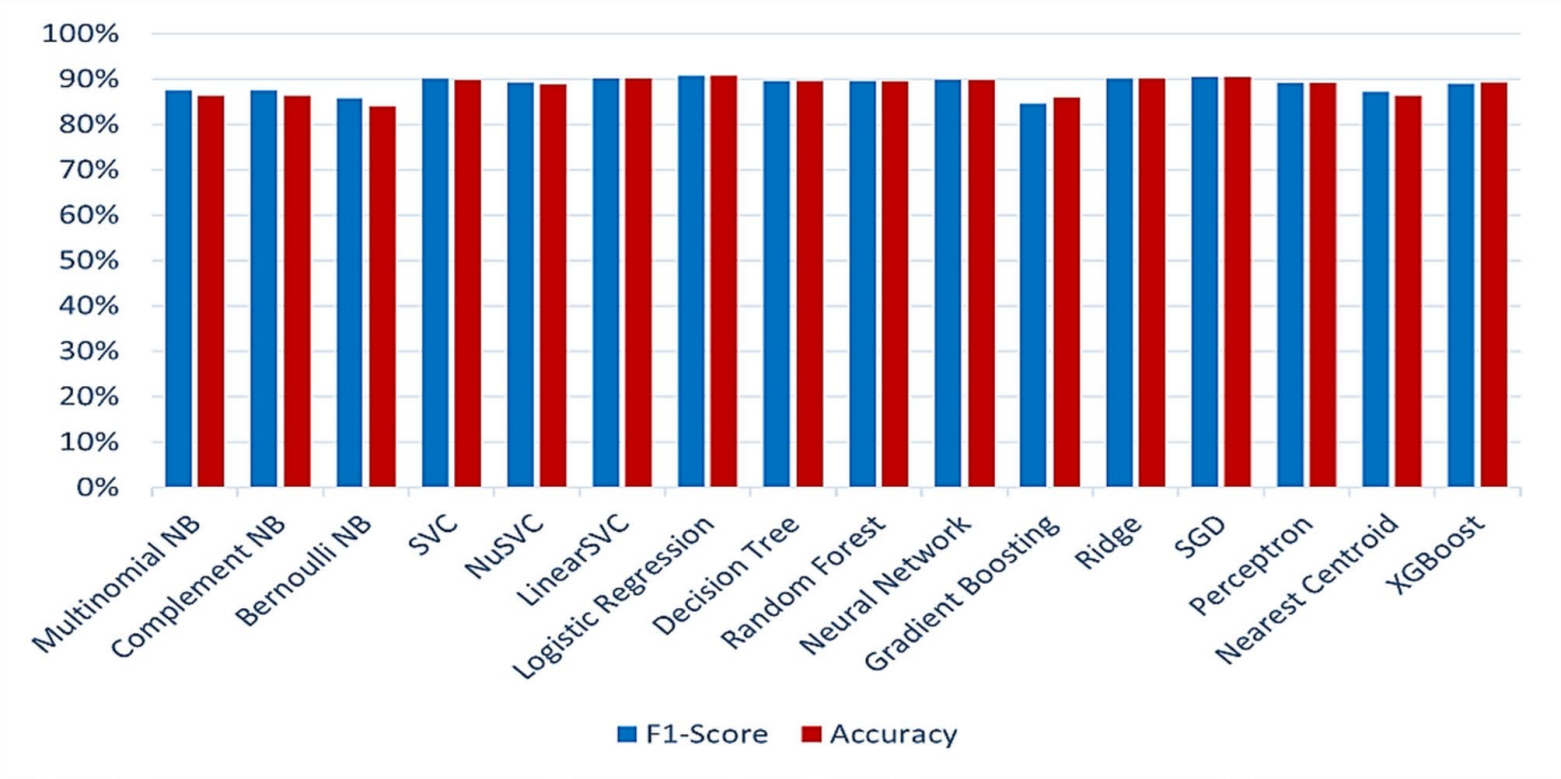
ML model evaluation (f1-score and accuracy) performance comparison using the BOW representation.

The text classification results obtained via the BOW representation are listed in Table 3; Fig. 10. These measures, such as precision, recall, the F1-score, and accuracy, are used with various classification algorithms.

**Table 3:** Results of the ML algorithms using the BOW representation.

	ML models (BOW)	Precision	Recall	F1-score	Accuracy
1	Multinomial NB	81.86%	93.89%	87.46%	86.35%
2	Complement NB	82.00%	93.76%	87.49%	86.40%
3	Bernoulli NB	77.61%	95.94%	85.81%	83.91%
4	SVC	89.06%	91.03%	90.04%	89.78%
5	NuSVC	87.41%	91.25%	89.29%	88.90%
6	LinearSVC	90.75%	89.83%	90.29%	90.20%
7	Logistic regression	**91.27%**	**90.32%**	**90.79%**	**90.71%**
8	Decision tree	90.22%	89.16%	89.69%	89.60%
9	Random forest	89.76%	89.48%	89.62%	89.49%
10	Neural network	90.27%	89.48%	89.87%	89.77%
11	Gradient boosting	96.09%	75.40%	84.50%	85.97%
12	Ridge	90.76%	89.59%	90.17%	90.09%
13	SGD	91.07%	90.13%	90.60%	90.51%
14	Perceptron	89.68%	88.82%	89.25%	89.15%
15	Nearest centroid	83.98%	90.54%	87.14%	86.45%
16	XGBoost	93.92%	84.54%	88.98%	89.38%

**Fig. 11:**
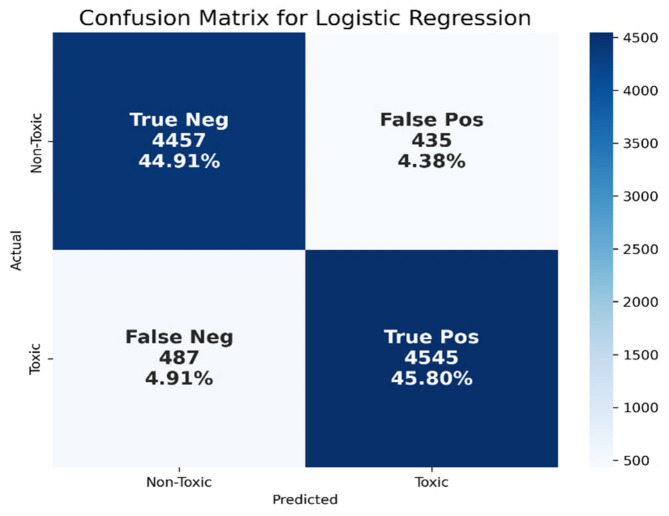
Confusion matrix for the logistic regression model using BOW features.

Table 3 shows that gradient boosting outperforms the other classifiers in terms of precision equal to 96.09%, the Bernoulli NB outperforms the other classifiers in terms of recall equal to 95.94%, and the logistic regression outperforms the other classifiers in terms of the F1-score and accuracy equal to 90.79% and 90.71%, respectively. However, there is a disparity between precision and recall, and between the F1-score and accuracy. The precision and recall do not consider false negative values (actual positives that were predicted as negatives), and the F1-score is a balance between these two metrics. Accuracy measures the correctness of the models across all classes (both true positives and true negatives). Therefore, we focus on the F1-score and accuracy, as we previously noted. It is easily deduced from Table 3; Fig. 10 that the classifier logistic regression performed better in terms of the F1-score and accuracy. The performance of logistic regression in terms of precision, recall, F1-score, and accuracy are 91.27%, 90.32%, 90.79%, and 90.71%, respectively. Figure 11 shows the confusion matrix for the logistic regression model using BOW features.

As shown in Fig. 11, this indicates that the model correctly identified 4,457 non-toxic tweets (TN) and 4,545 toxic tweets (TP), while misclassifying 435 non-toxic tweets as toxic (FP) and 487 toxic tweets as non-toxic (FN). The relatively high TP rate (45.80%) demonstrates BoW’s effectiveness at detecting explicit toxicity, particularly in tweets that contain frequent and unambiguous toxic vocabulary. For instance, in the example “أنت إنسان فاشل وكل الناس تكرهك” (“You’re a failure and everyone hates you”), BoW correctly predicted toxicity by matching the terms “فاشل” (“failure”) and “تكرهك” (“hates you”) with toxic words in its learned vocabulary. Similarly, the model succeeded in identifying polite and respectful language such as “أحب العمل الجماعي وأحترم كل الآراء” (“I love teamwork and respect all opinions”) as non-toxic, contributing to its 44.91% TN rate. However, the BoW model struggled to capture contextual and nuanced language, resulting in 4.91% FN and 4.38% FP. Regarding the FN, the tweet “إنت تثير الاشمئزاز بتصرفاتك لكن مش مهم” (“You disgust people with your actions, but whatever”) was incorrectly labeled as non-toxic. The model likely missed the toxic intent because it is conveyed implicitly through tone and phrasing rather than direct lexical cues. In terms of FP, a neutral tweet such as “النقاش في هذا الموضوع حساس ويحتاج إلى احترام” (“This topic is sensitive and requires respectful discussion”) was misclassified as toxic, possibly due to the word “حساس” (“sensitive”), which may have frequently co-occurred with toxic tweets during training, misleading the model. These limitations highlight BoW’s vulnerability to contextual ambiguity and its reliance on isolated word frequencies without semantic understanding.

### TF-IDF representation

**Fig. 12:**
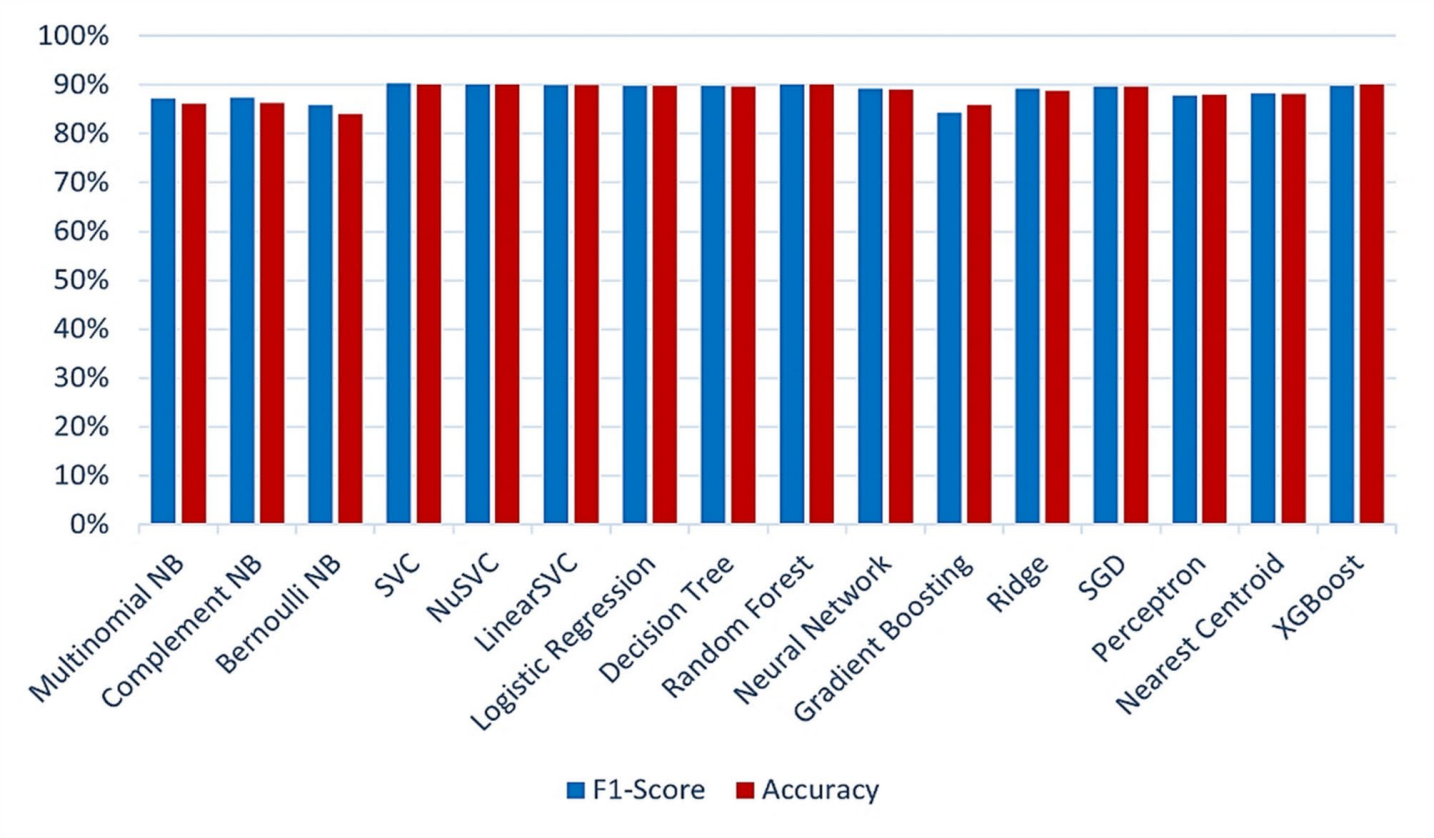
ML model evaluation (f1-score and accuracy) performance comparison using the TF-IDF representation.

Table 4; Fig. 12 show the results for the classification task when the TF-IDF representation is used.

**Table 4:** ML algorithm results using the TF-IDF representation.

	ML models (TF-IDF)	Precision	Recall	F1-score	Accuracy
1	Multinomial NB	81.42%	93.93%	87.22%	86.05%
2	Complement NB	81.99%	93.40%	87.32%	86.25%
3	Bernoulli NB	77.61%	95.94%	85.81%	83.91%
4	SVC	**90.44%**	**89.96%**	**90.20%**	**90.10%**
5	NuSVC	90.49%	89.83%	90.16%	90.06%
6	LinearSVC	90.81%	89.18%	89.98%	89.93%
7	Logistic Regression	90.14%	89.51%	89.82%	89.72%
8	Decision Tree	90.12%	89.50%	89.81%	89.70%
9	Random Forest	90.97%	89.23%	90.09%	90.05%
10	Neural Network	89.14%	89.10%	89.12%	88.97%
11	Gradient Boosting	96.75%	74.60%	84.24%	85.85%
12	Ridge	87.66%	90.65%	89.13%	88.79%
13	SGD	89.90%	89.51%	89.71%	89.58%
14	Perceptron	90.04%	85.70%	87.82%	87.94%
15	Nearest Centroid	88.77%	87.61%	88.18%	88.09%
16	XGBoost	94.16%	85.81%	89.79%	90.09%

**Fig. 13:**
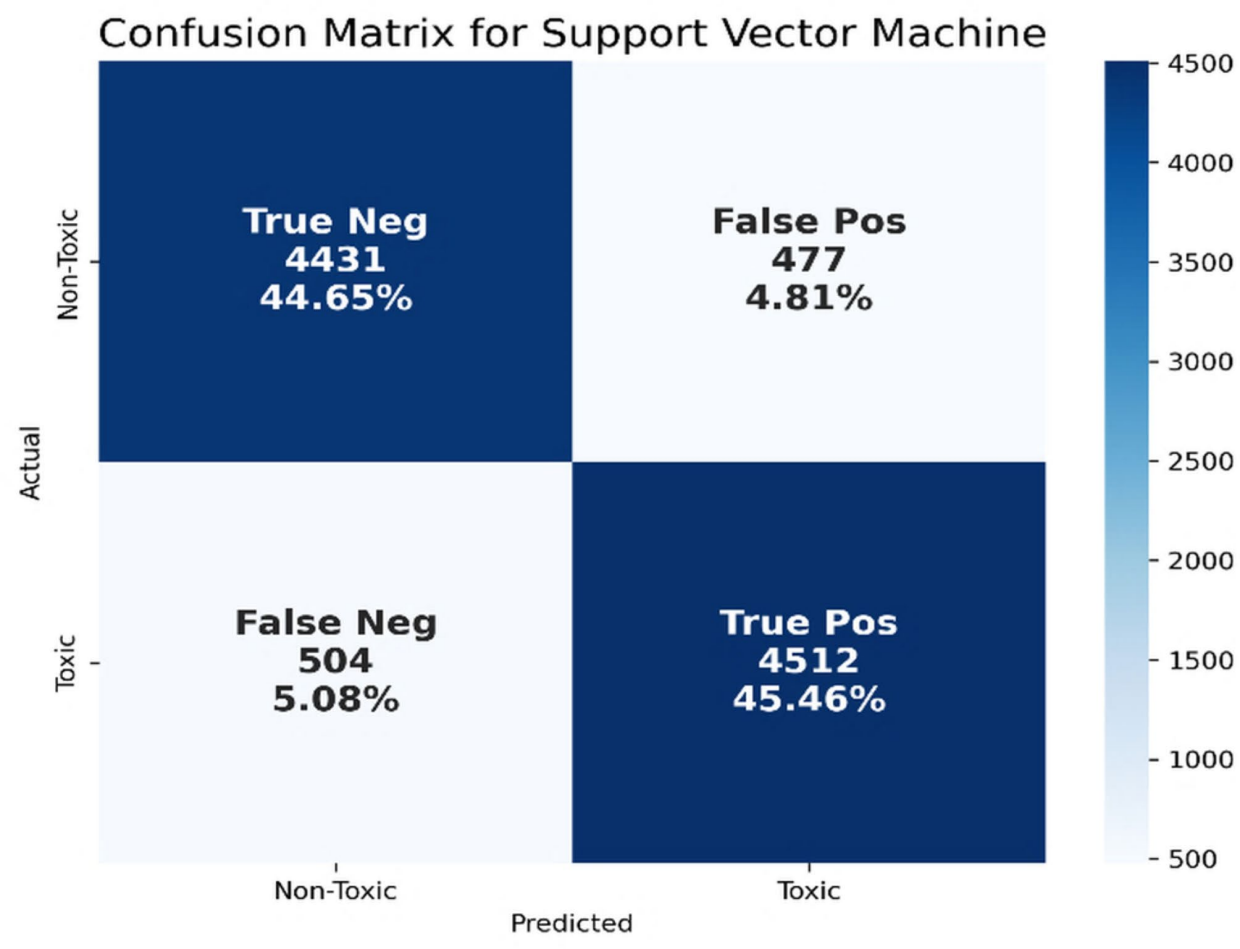
Confusion matrix for the SVM model using TF-IDF features.

Table 4; Fig. 12 show that the SVM classifier had the best performance with respect to the F1-score and accuracy. The performance of SVC in terms of precision, recall, F1-score, and accuracy are 90.44%, 89.96%, 90.20%, and 90.10%, respectively. Figure 13 shows the confusion matrix for the SVM model using TF-IDF features.

As shown in Fig. 13, this indicates that the model correctly classified 4,431 non-toxic tweets (TN) and 4,512 toxic tweets (TP), while misclassifying 477 non-toxic tweets as toxic (FP) and 504 toxic tweets as non-toxic (FN). The high TP rate (45.46%) indicates the model’s capacity to identify toxic content, especially in tweets with distinctive, high-weight toxic terms. For example, in “هؤلاء الجهلة لا يستحقون الكلام معنا” (“These ignorant people don’t deserve to talk to us”), the words “الجهلة” (“the ignorants”) and “لا يستحقون” (“they don’t deserve”) are rare but strongly associated with toxic language, making them highly weighted under TF-IDF and contributing to correct classification. Similarly, the model effectively recognized non-toxic content, such as “الجو هادئ اليوم والمكان مريح” (“The weather is calm today and the place is peaceful”), which contains frequent, low-weight neutral terms, leading to a solid TN rate of 44.65%. However, the model struggled with contextually ambiguous expressions, leading to 4.81% FP and 5.08% FN. A typical FP case involved the tweet “هذا المستخدم يكرر منشوراته كثيرًا” (“This user repeats their posts a lot”), which, despite being non-toxic, was flagged as toxic. This likely occurred because words like “يكرر” (“repeats”) may appear in toxic tweets criticizing spammers, thus being assigned a high weight in TF-IDF. On the other hand, the tweet “أشباه الرجال لا مكان لهم بيننا” (“Men like you have no place among us”) was misclassified as non-toxic. Despite its clear toxic meaning, the lack of frequent toxic terms or its formal phrasing might have caused TF-IDF to underweight its severity, highlighting the model’s limitations in detecting implicit toxicity.

### FastText representation

The results for this text classification using the FastText representation are shown in Table 5.

**Table 5:** Results of fasttext (default form) when fasttext is used as a feature representation.

FastText model	Precision	Recall	F1-score	Accuracy
FastText (default form)	90.26%	90.26%	90.26%	90.26%

**Fig. 14:**
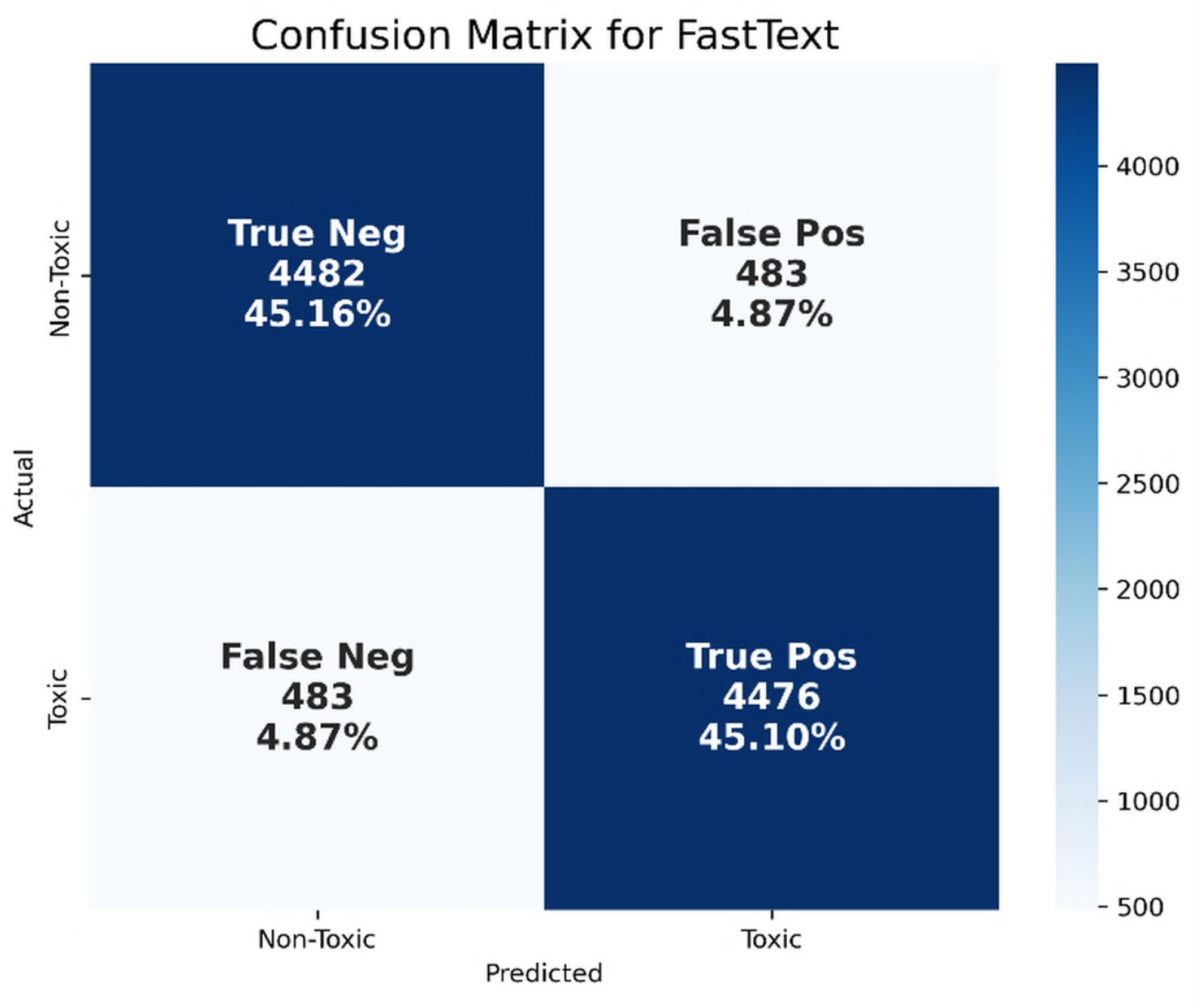
Confusion matrix for the FastText (default form) model.

As mentioned in Table 5, the FastText model (Default Form) was used for performance evaluation in terms of precision, recall, F1-score, and accuracy, which were 90.26%, 90.26%, 90.26%, and 90.26%, respectively. Figure 14 shows the confusion matrix for the FastText (Default Form) model.

As shown in Fig. 14, this indicates that the model correctly identified 4,482 non-toxic tweets (TN) and 4,476 toxic tweets (TP), while it misclassified 483 non-toxic tweets as toxic (FP) and 483 toxic tweets as non-toxic (FN). The nearly equal TP and TN rates (45.10% and 45.16%, respectively) suggest that FastText is generally reliable in both recognizing toxic and non-toxic content. FastText’s use of subword embeddings enables it to learn word structures and handle spelling variations, allowing for effective generalization across dialects and inflected forms. This also helps the model capture meaningful patterns in morphologically rich languages such as Arabic. For instance, the toxic tweet “اخرس ولا تعلق، وجودك غير مرحب به” (“Shut up and don’t comment, you’re not welcome here”) includes multiple aggressive phrases such as “اخرس” (“shut up”) and “غير مرحب” (“not welcome”) which FastText correctly identifies as toxic based on their frequent occurrence in hostile contexts during training. On the other hand, it successfully recognized the non-toxic tweet “كل شخص لديه رأي ويجب احترامه” (“Everyone has an opinion, and it should be respected”) due to its calm and respectful tone, captured by key terms like “رأي” and “احترام”. Despite this balanced performance, the model still produced symmetrical error rates (4.87%) in both classes. FP occurred when neutral or mildly critical statements were misclassified as toxic. For example, the tweet “حديثك غريب لكن ممتع” (“Your talk is strange but enjoyable”) was incorrectly labeled as toxic, likely because the word “غريب” (“strange”), although benign in this context, may frequently appear in sarcastic or negative tweets, leading FastText to associate it with toxicity. Conversely, FN involved implicit, subtle, or indirect expressions of toxicity. The tweet “أمثالك يجب حظرهم لأنكم بلا أخلاق” (“People like you should be banned because you have no morals”) was misclassified as non-toxic despite its strongly judgmental tone. This type of error reveals FastText’s limitations in modeling the nuanced semantics and deeper contextual intent, especially in the absence of overtly offensive terms.

### BERT representation

**Fig. 15:**
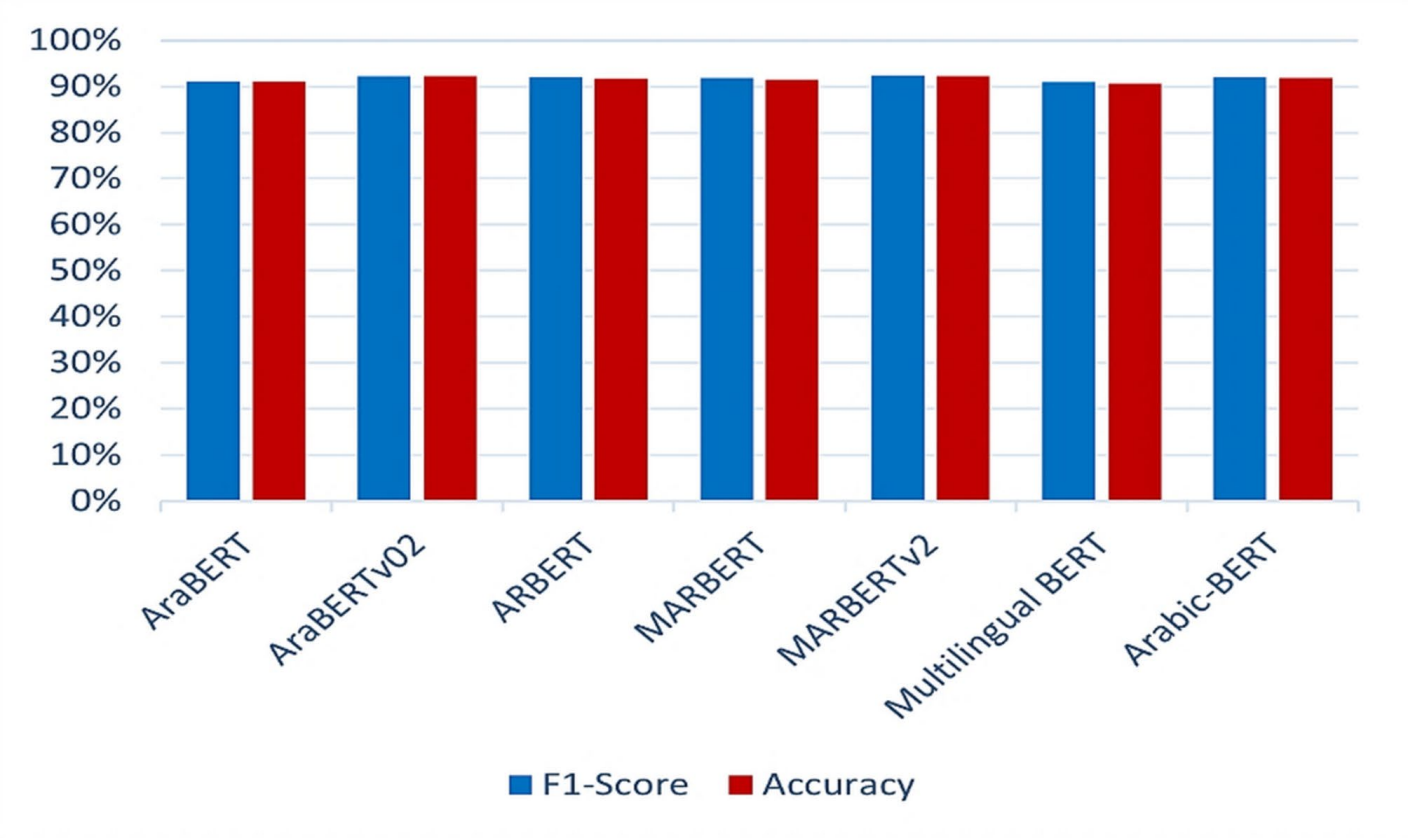
TL model evaluation (f1-score and accuracy) performance comparison when the BERT representation is used.

The classification results obtained via the BERT representation model are presented in Table 6; Fig. 15.

**Table 6:** TL algorithm results when the BERT representation is used.

	TL models (BERT)	Precision	Recall	F1-score	Accuracy
1	AraBERT	90.85%	91.53%	91.19%	91.03%
2	AraBERTv02	91.95%	92.60%	92.27%	92.13%
3	ARBERT	89.16%	94.97%	91.97%	91.59%
4	MARBERT	90.16%	93.51%	91.81%	91.54%
5	MARBERTv2	**91.06%**	**93.85%**	**92.43%**	**92.21%**
6	Multilingual BERT	89.71%	92.09%	90.89%	90.64%
7	Arabic-BERT	90.92%	93.23%	92.06%	91.85%

**Fig. 16:**
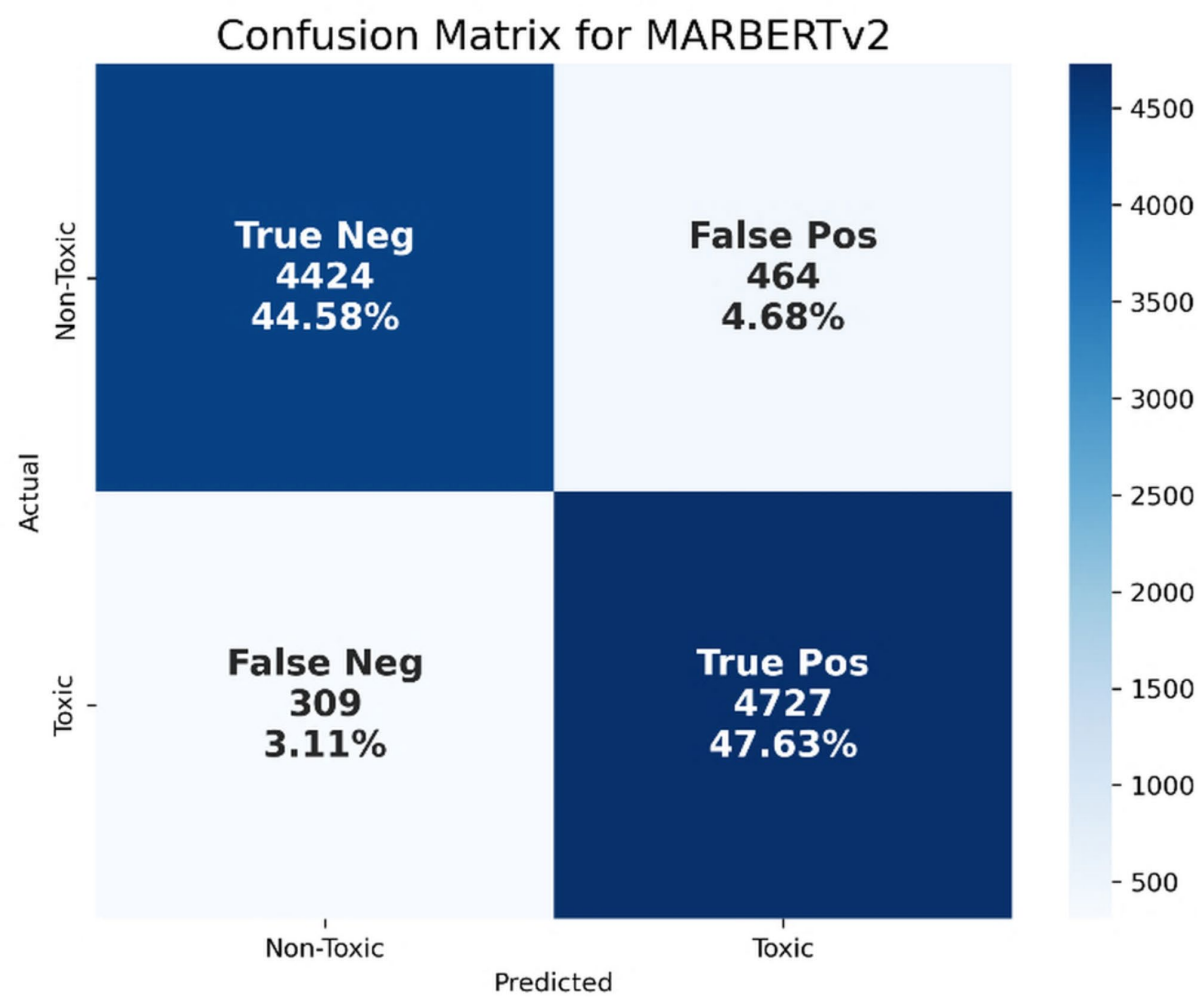
Confusion matrix for the MARBERTv2 model using BERT features.

From Table 6; Fig. 15, we conclude that MARBERTv2 achieved the highest overall performance among all evaluated BERT-based models, with precision, recall, F1-score, and accuracy reaching 91.06%, 93.85%, 92.43%, and 92.21%, respectively. This superior performance can be attributed to MARBERTv2’s pretraining on billions of Arabic tweets, which allows it to effectively handle dialectal diversity, informal language, and noisy user-generated content. In contrast, models such as AraBERT, AraBERTv02, and ARBERT are primarily trained on MSA from formal domains, limiting their ability to capture colloquial expressions and dialects. Multilingual BERT, while multilingual, lacks Arabic-specific optimization and struggles with dialect-rich input. Although Arabic-BERT and MARBERT incorporate some dialectal data, they do not match MARBERTv2’s scale or coverage. Additionally, MARBERTv2’s tokenizer is optimized for the lexical variability of social media, enabling better recognition of out-of-vocabulary terms, colloquial expressions, and spelling variants. Figure 16 shows the confusion matrix for the MARBERTv2 model using BERT features.

As shown in Fig. 16, this indicates that the model correctly classified 4,424 non-toxic tweets (TN) and 4,727 toxic tweets (TP), while misclassifying 464 non-toxic tweets as toxic (FP) and only 309 toxic tweets as non-toxic (FN). The notably high TP rate (47.63%) highlights MARBERTv2’s strong ability to detect toxic language, including sarcasm, figurative speech, and dialectal variation. For example, the tweet “أنتم سرطان هذا الوطن، مكانكم في القمامة” (“You are the cancer of this country, you belong in the trash”) demonstrates toxic intent through metaphor and insult. Unlike traditional models, MARBERTv2 successfully interprets such indirect yet clearly aggressive language due to its deep contextual and semantic understanding. Its similarly high TN rate (44.58%) also reflects strong performance in identifying non-toxic content. A tweet such as “أعتقد أننا نختلف في وجهات النظر وهذا طبيعي” (“I think we differ in our views and that’s normal”) conveys respectful disagreement, which MARBERTv2 appropriately classifies as non-toxic. The FP rate (4.68%) is relatively low, but errors still occur when the model misinterprets benign expressions such as sarcasm or covert hostility. For instance, “انت أسطورة في شغلك، الكل بيحترمك” (“You’re a legend in your work, everyone respects you”) was flagged as toxic, potentially due to the model’s sensitivity to exaggerated praise that may appear sarcastic in toxic contexts. On the other hand, the relatively low FN rate (3.11%) shows MARBERTv2’s strength in minimizing under-detection of toxicity. Nonetheless, it occasionally misses more subtle forms of toxic speech, such as in “وجودك هو السبب في كل المصايب اللي بنعيشها” (“Your presence is the reason behind all the disasters we live through”). Despite the implicit blame and emotional tone, the lack of explicit insult may lead the model to misclassify it as non-toxic. In summary, MARBERTv2 with BERT-based embeddings offers superior performance in Arabic toxicity detection. Its ability to model rich context and handle diverse linguistic forms, particularly across dialects and figurative speech, makes it especially effective in capturing both overt and nuanced toxic behavior, with fewer misclassifications compared to traditional models.

**Fig. 17:**
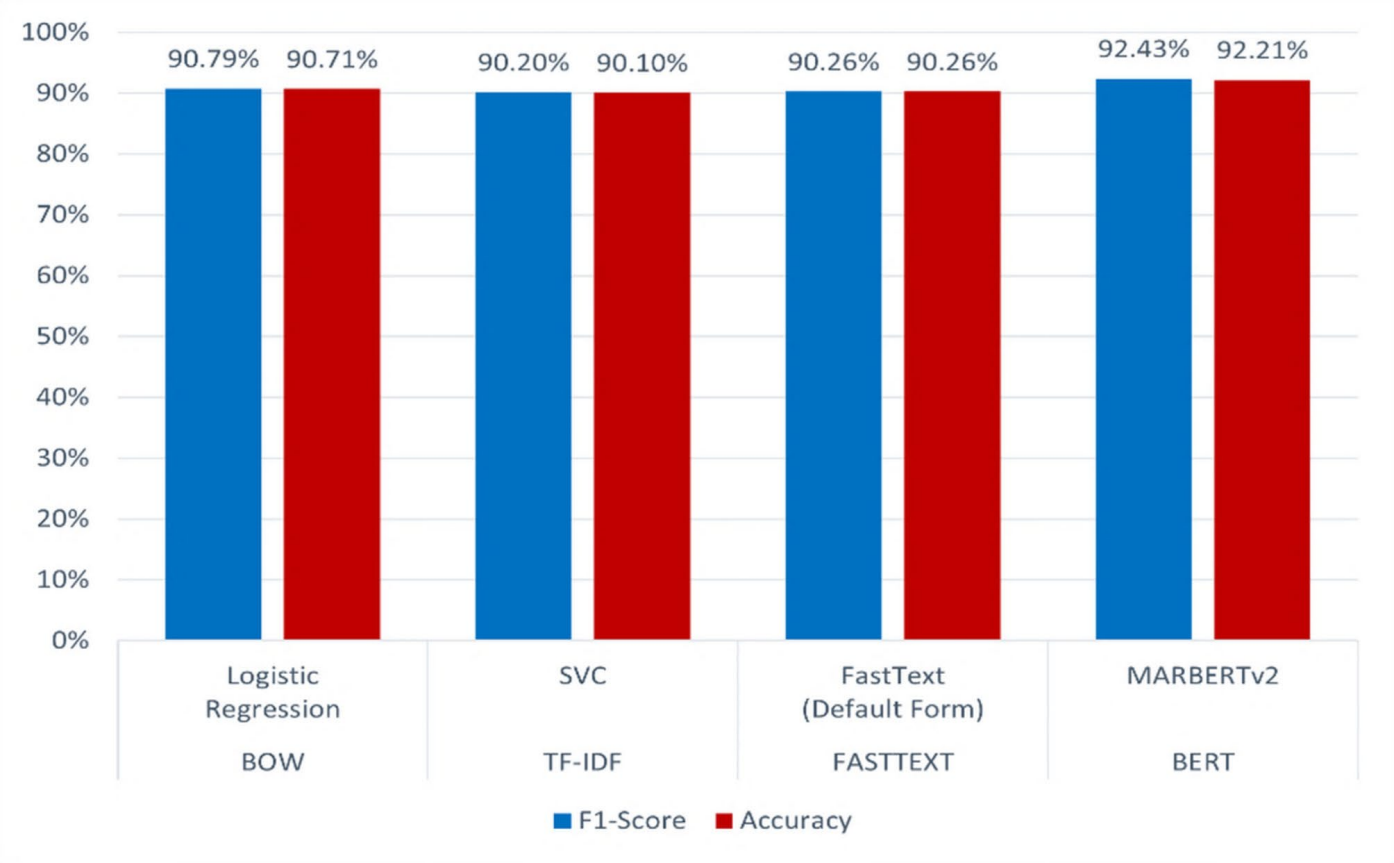
Best Models evaluation (f1-score, and accuracy) performance comparison.

The best classification models evaluated by using feature representations are shown in Table 7; Fig. 17.

**Table 7:** Comparison of the performance of the best models (F1-score and accuracy).

	Feature representations	Best models	F1-score	Accuracy
1	BOW	Logistic Regression	90.79%	90.71%
2	TF-IDF	SVC	90.20%	90.10%
3	FASTTEXT	FastText (Default Form)	90.26%	90.26%
4	BERT	MARBERTv2	**92.43%**	**92.21%**

In summary, the results are outlined in Table 7; Fig. 17. The MARBERTv2 classifier with BERT representation is the best architecture for Arabic toxicity classification in online social media (Twitter) in terms of the F1-score, with accuracies of 92.43% and 92.21%, respectively.

To validate the statistical significance of performance differences among the best-performing classification models, we conducted a series of statistical tests based on the F1-score results shown in Table 7. ANOVA (Analysis of Variance) is a widely used statistical method for assessing whether there are statistically significant differences between the means of two or more independent groups^71^. Table 8 summarizes the statistical tests applied to evaluate the significance of differences in F1-scores among the top classification models on the proposed dataset.

**Table 8:** Statistical tests applied to evaluate the significance of differences in F1-scores among the best-performing classification models.

Test	Level of significance (alpha α)	*P*-Value
Shapiro–Wilks normality test	0.05	0.8445
One-Way Anova test	0.05	0.0009

**Fig. 18:**
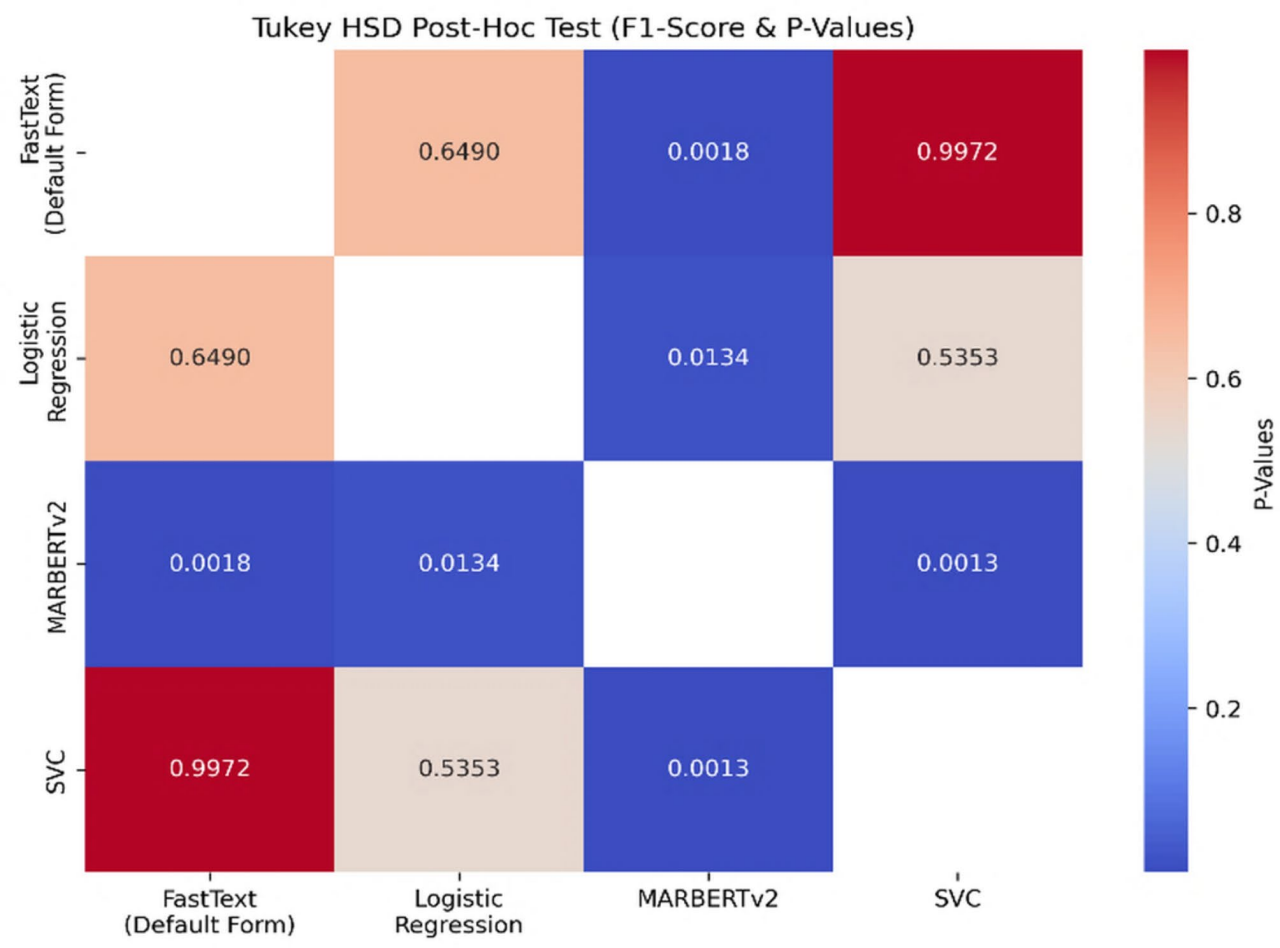
Heatmap of pairwise p-values from the Tukey HSD Post-Hoc test.

As shown in Table 8, the Shapiro–Wilk test was first applied to assess the normality assumption, a prerequisite for using parametric tests such as ANOVA. The resulting p-value (0.8445) exceeded the significance threshold α = 0.05, indicating that the distribution of F1-scores follows a normal (Gaussian) distribution^72^. This confirmed the appropriateness of conducting parametric statistical analysis. Subsequently, a one-way ANOVA test was performed to determine whether there were statistically significant differences in F1-scores among the models. The ANOVA yielded a p-value of 0.0009, which is below the α level of 0.05, leading to the rejection of the null hypothesis. This confirms that at least one model’s performance differs significantly from the others. To identify which specific models exhibited significant differences, the Tukey HSD (Honestly Significant Difference) post-hoc test was applied^73^. This test is used to compare all pairs of models to determine which differences are statistically significant, while ensuring the reliability of results when multiple comparisons are involved. The results showed that MARBERTv2 significantly outperformed all other models (*p* < 0.05), while no statistically significant differences were found among the remaining models. These pairwise significance levels and differences are illustrated in Fig. 18, which presents a heatmap summarizing the p-values and statistical significance of each model comparison.

Table 9 shows the F1-score and accuracy evaluation performance comparison between the mentioned evaluation classifiers on our proposed Arabic toxicity dataset and other available Arabic datasets mentioned in the related work section.

**Table 9:** Arabic toxicity dataset performance comparison.

	Study/dataset	F1-score (%)	Accuracy (%)
1	Proposed dataset	**92.43**	**92.21**
2	Harbaoui, A. et al.^13^	48	-
3	Lee, N. et al.^17^	82	-
4	Mazari, A. C. and Kheddar, H.^21^	75.8	73.6
5	Harrag, F. et al.^22^	78	81
6	Alrashidi, B. et al.^23^	91	89
7	Al-Hassan, A. and Al-Dossari, H.^26^	73	-
8	Badri, N. et al.^27^	82	-
9	Husain, F. and Uzuner, O.^30^	87	87
10	Masadeh, M. et al.^32^	79	79
11	Boulouard, Z. et al.^34^	82	82
12	Muaad, A. Y. et al.^36^	90	91
13	Mohdeb, D. et al.^38^	57	68
14	Alhayan, F. et al.^39^	84	85
15	Tashtoush, Y. et al.^40^	91.9	91.9

From the comparison in terms of the F1-score and accuracy metrics with the other datasets, as shown in Table 9, it is evident that our proposed Arabic toxicity dataset performed the best.

## Limitations

While our results demonstrate high accuracy and robustness in Arabic toxicity detection, this study has several limitations that should be acknowledged. First, the dataset was constructed using a predefined list of toxic keywords, which may introduce selection bias, favoring overtly toxic expressions while potentially missing subtle or context-dependent toxicity. Second, our data collection was limited to Twitter, which may affect the generalizability of the models to other platforms such as Facebook or YouTube, where language use, structure, and community norms may differ. Third, although this study focuses exclusively on toxic tweet classification in the Arabic language, code-mixed content (e.g., Arabic mixed with English or Franco-Arabic) and dialect switching, both prevalent in real-world social media, were not explicitly addressed. Therefore, the findings may not be directly applicable to other languages or multilingual contexts, limiting broader generalizability. Despite these limitations, the study demonstrates promising results, with the MARBERTv2 model with BERT representation outperforming other models, highlighting its advancement in Arabic toxic tweet classification and emphasizing the importance of addressing toxicity while considering diverse Arabic dialects and cultures on Twitter platform.

## Conclusion

This paper offers a thorough technique for dealing with toxic and harmful content on social media sites, with an emphasis on the classification of Arabic toxic tweets. We created a dataset of 49,620 Arabic tweets that contain a variety of various Arabic dialects from different regions that have been manually annotated by using the extensive knowledge of five native and fluent Arabic speakers and linguists into two binary classes: Toxic and Non-Toxic. These two classes are balanced. This study highlights how important model selection and contextual factors are in correctly classifying toxic and harmful tweets. We investigated a variety of machine and transfer learning models and word embedding representations and fine-tuned optimized pre-trained models, assessing their effectiveness performance via various metrics. Sixteen machine learning algorithms (e.g., Multinomial NB, Complement NB, Bernoulli NB, SVC, NuSVC, LinearSVC, Logistic Regression, Decision Tree, Random Forest, Neural Network, Gradient Boosting, Ridge, SGD, Perceptron, Nearest Centroid, and XGBoost), the FastText (as its default form) classification model, and seven transfer learning architectures (e.g., AraBERT, AraBERTv02, ARBERT, MARBERT, MARBERTv2, Multilingual BERT, and Arabic-BERT) were applied to calculate the dataset’s evaluation performance. Several feature representations (e.g., bag of words (BoW), term frequency–inverse document frequency (TF-IDF), FastText, and bidirectional encoder representations from transformers (BERT)) were used with the classification algorithms. The results demonstrated that the MARBERTv2 model with BERT as a text representation has extraordinary potential, as it continuously outperforms competing models in performance, with outstanding F1-scores and accuracy evaluation metrics of 92.43% and 92.21%, respectively. In future work, we aim to expand our dataset by collecting content from diverse platforms and regions to include more Arabic dialects and cultural variations. We also plan to adopt multiclass classification schemes instead of binary labeling to better capture varying degrees and types of toxicity. To enhance model performance, we will explore additional deep learning architectures, utilize larger-scale transfer learning techniques, and apply varied embedding models such as word2vec, ELMo, GPT, and T5. Furthermore, we intend to investigate multimodal approaches by incorporating textual, audio, and visual data, enabling broader application to platforms like Facebook, Instagram, YouTube, and TikTok. Another promising direction is cross-lingual learning, where large-scale English toxicity datasets or multilingual pre-trained models can be leveraged to improve Arabic performance. Finally, incorporating context-aware conversation modeling can help in detecting more subtle forms of toxicity, such as sarcasm or indirect insults, or temporally dependent toxic intent, by considering dialogue context.

## Data Availability

The data that support the findings of this study are not publicly available due to reasons of sensitivity and privacy requirements and are available from the corresponding author upon reasonable request. A comparable dataset can also be generated following the methodology described in this study, including data acquisition, preprocessing, and annotation steps.
